# A novel inhibitor L755507 efficiently blocks c-Myc–MAX heterodimerization and induces apoptosis in cancer cells

**DOI:** 10.1016/j.jbc.2021.100903

**Published:** 2021-06-23

**Authors:** Ashutosh Singh, Ankur Kumar, Prateek Kumar, Namyashree Nayak, Taniya Bhardwaj, Rajanish Giri, Neha Garg

**Affiliations:** 1 School of Basic Sciences and Advanced Materials Research Center, Indian Institute of Technology Mandi, Mandi, Himachal Pradesh, India; 2 Department of Medicinal Chemistry, Institute of Medical Sciences, Banaras Hindu University, Varanasi, Uttar Pradesh, India

**Keywords:** c-Myc–MAX heterodimer, protein–protein interaction, virtual screening, compound inhibitors, drug discovery, bHLH-LZ, basic helix–loop–helix leucine zipper, GEPIA, Gene Expression Profiling Interactive Analysis, ITC, isothermal titration calorimetry, PDB, Protein Data Bank, Pol_B_Sul, polymyxin B sulfate, PPI, protein–protein interaction, qPCR, quantitative PCR, RMSFs, root mean square fluctuations, Trp, tryptophan

## Abstract

c-Myc is a transcription factor that plays a crucial role in cellular homeostasis, and its deregulation is associated with highly aggressive and chemotherapy-resistant cancers. After binding with partner MAX, the c-Myc–MAX heterodimer regulates the expression of several genes, leading to an oncogenic phenotype. Although considered a crucial therapeutic target, no clinically approved c-Myc-targeted therapy has yet been discovered. Here, we report the discovery *via* computer-aided drug discovery of a small molecule, L755507, which functions as a c-Myc inhibitor to efficiently restrict the growth of diverse Myc-expressing cells with low micromolar IC_50_ values. L755507 successfully disrupts the c-Myc–MAX heterodimer, resulting in decreased expression of c-Myc target genes. Spectroscopic and computational experiments demonstrated that L755507 binds to the c-Myc peptide and thereby stabilizes the helix–loop–helix conformation of the c-Myc transcription factor. Taken together, this study suggests that L755507 effectively inhibits the c-Myc–MAX heterodimerization and may be used for further optimization to develop a c-Myc-targeted antineoplastic drug.

The protooncogene *c-myc* (referred to here as Myc), a basic helix–loop–helix leucine zipper (bHLH-LZ) transcription factor, is involved in the regulation of multiple cellular functions including cell growth, proliferation, and apoptosis, while blocking the differentiation (1). Myc dimerizes with a relatively small partner MAX, also a bHLH-LZ protein, and regulates the expression of nearly 15% of the entire human genome (2). The Myc–MAX heterodimer specifically binds to the palindromic enhancer box sequences or E-box present at the promoter, which then prompts the recruitment of chromatin remodeling complexes (3) and transcription initiation machinery (4) and thereby modulates the transcription of the target genes. The human Myc gene is located on chromosome 8 at locus 8q24.21 and is under tight control both at the transcriptional and translational levels (5). However, a glitch in the regulation process leads to Myc's aberrant expression because of chromosomal translocation, insertional mutation, and gene amplification. The deregulated expression of Myc is linked to approximately 75% of human cancers (6), showing aggressive nature and low sensitivity toward available chemotherapy (7).

Although Myc plays a crucial role in regulating the vital cellular functions of the healthy cells, the pioneer study on the preclinical model of Ras-induced lung adenocarcinoma has shown that Myc inhibition leads to rapid erosion in the tumor mass with slight and reversible effect on the surrounding healthy tissues (8). This study, along with Myc's involvement in many cancers, suggests it a viable therapeutic target for developing novel antineoplastics. Several indirect and direct approaches have been used for endogenous targeting of Myc, which involve upstream Myc regulators (9, 10), Myc–MAX dimerization (11, 12), Myc–MAX dimer–DNA interaction (13, 14), G-quadruplex stabilizers (15, 16), and siRNAs (17, 18). Targeting the Myc–MAX interaction has emerged as the preferred strategy for identifying new inhibitors that explicitly modulate Myc's regulatory functionality. The initial attempt leads to identifying 10058-F4, 10074-A4, and 10074-G5 as a dimerization inhibitor with low potency toward Myc-overexpressing cells with mid-micromolar IC_50_ values (12). Till now, several structurally diverse small-molecule inhibitors have been reported, but they lack *in vivo* potency and appropriate pharmacokinetic properties (6, 11, 19). The bHLH-LZ domain of the two partner proteins is responsible for the heterodimerization and the formation of the DNA-binding domain. The disordered bHLH-LZ domain undergoes ordered transformation after dimerization *via* adopting a stable helical conformation (20, 21). Myc and MAX interaction involves a large flat interface area of ∼3200 Å^2^ that lacks appropriate and uncharacterized small-molecule binding sites (22, 23). Owing to the disordered nature of Myc and lack of well-defined binding sites, the Myc–MAX dimerization is defined as “undruggable” (24, 25). Owing to these characteristics, targeting Myc and MAX for therapeutic intervention *via* small molecules makes it a challenging job.

Progressing to Myc targeting research, we performed a structure-based drug discovery of bioactive molecules on the druggable site on the Myc bHLH domain identified from the computational algorithm. The top hit molecule screened from the database binds to the Myc bHLH domain, resulting in the disruption of Myc–MAX dimerization. The cell-based assays proved the compound's substantial potency to restrict the Myc-driven oncogenic phenotypes and inhibit Myc's transcriptional regulation. Thus, our study reported a bioactive compound as a potent inhibitor of the Myc–MAX heterodimer.

## Results

### Identification of potential druggable site and discovery of bioactive molecules that target the bHLH domain

**Figure 1:**
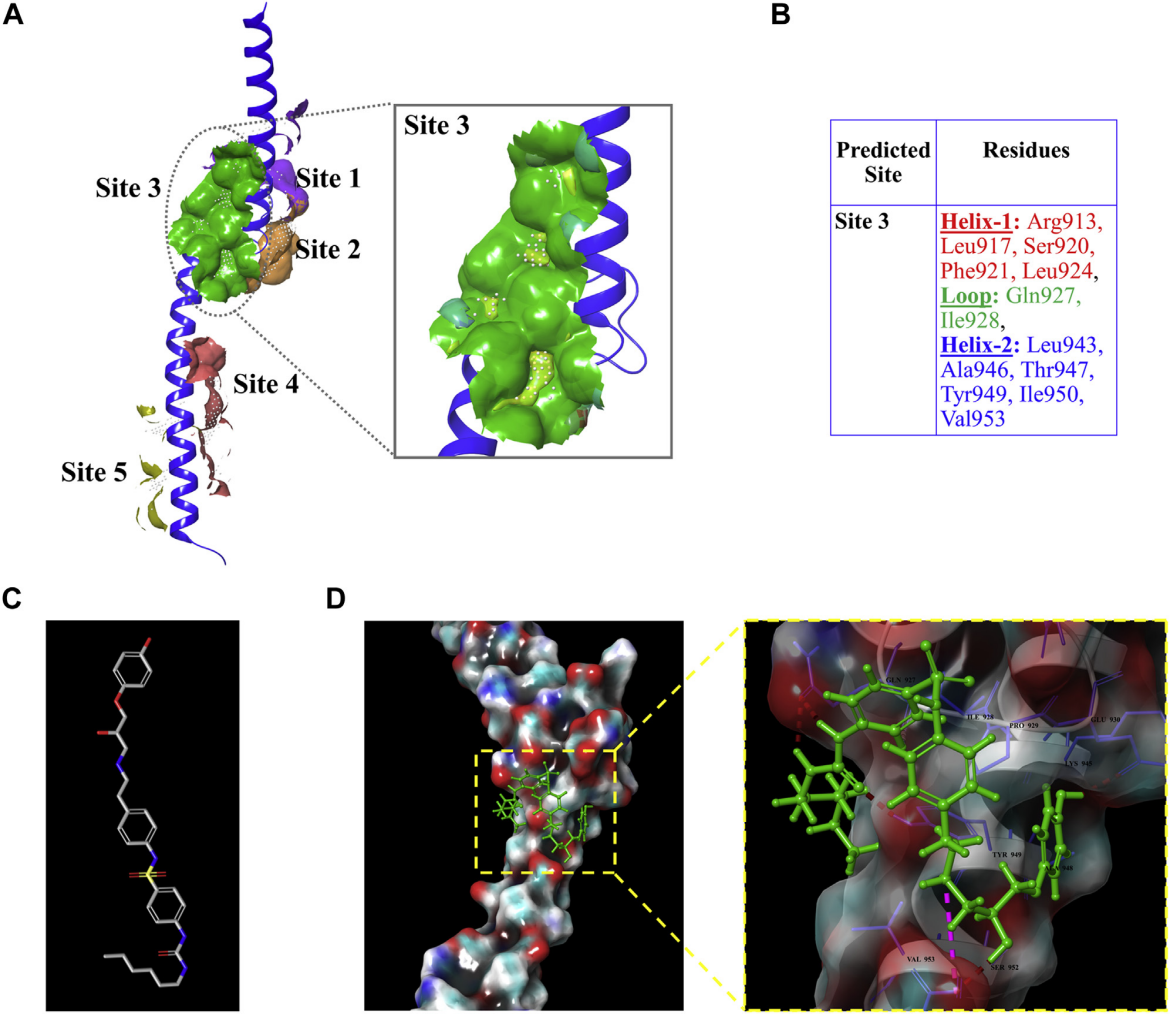
**High-throughput virtual screening aided in the discovery of hit bioactive compounds against oncogenic Myc.***A*, depiction of five active sites predicted through the Sitemap tool. Site 3 is represented as a *green solid surface* in a magnified view and was chosen to screen bioactive compounds from the Selleckchem database. *B*, list of amino acid residues shaped in site 3 (helix 1, loop region, and helix 2) of the bHLH domain. *C*, chemical structure of L755507. *D*, the potential binding poses and ligand molecular interactions of L755507 with the identified site 3 residues of Myc. bHLH-LZ, basic helix–loop–helix leucine zipper.

The Myc–MAX heterodimer interface is flat, relatively large, and lacks appropriate groves that facilitate small molecules' binding. Therefore, the crystal structure of Myc–MAX heterodimer bound to DNA (Protein Data Bank [PDB] ID: 1NKP (26)) was selected to identify a plausible binding site for bioactive molecules. The robust algorithm of the Sitemap module identified five potential binding sites with more site points and exposure to the solvent (Table S3). We chose site 3 to screen bioactive molecules as it justifies all the characteristics of a druggable site *viz.* site volume, solvent accessibility, and hydrophobicity (27). Site 3 is located just above the fork formed by the bHLH domain and lies well in the Myc–MAX heterodimer interface. Site 3 is defined by the residues from helix 1 *viz.* Arg913, Leu917, Ser920, Phe921, and Leu924, loop region *viz.* Gln927 and Ile928, and helix 2 *viz.* Leu943, Ala946, Thr947, Tyr949, Ile950, and Val953 (Fig. 1*A*). The functional relevance of amino acid residues residing in site 3 can be proven by its involvement in the molecular interaction with reported Myc inhibitors (28, 29, 30).

The employment of structure-based drug design is a distinctive approach in discovering new inhibitors or drug molecules in less time and in a cost-effective manner. And therefore, virtual screening was used to screen the database of 12,433 bioactive compounds from the Selleckchem library to discover hit molecules potentially disrupting Myc–MAX dimerization. After docking with the Glide XP method, 33,768 binding poses of the compounds were produced with Myc with a docking score ranging from −12 to −2 kcal/mol. All the compounds having a docking score of more than the cut-off value of −5 kcal/mol were selected for binding energy calculation using the molecular mechanics–generalized born surface area method through the Prime module (Fig. S1). Considering the docking score and binding energy, we performed the visual inspection of all the top compounds for their interaction with amino acid residues shaping site 3. Moreover, the known Myc inhibitors from the literature, which are reported to bind to the residues shaped in site 3 (28, 30), were chosen as a standard control. The virtual screening data showed that the binding affinity of the top hit molecules from the bioactive library was far superior to the known Myc inhibitors, which was reflected from the high docking score and binding energy (Figs. S1 and S2, *B*–*D* and Table S4).

From the virtually screened compounds, L755507 and polymyxin B sulfate (Pol_B_Sul) exhibited higher affinity and binding energy because of their multiple noncovalent interactions with the residues of site 3 (Fig. 1*D* and Fig. S2*A*). In the case of L755507, the Glu930 residue of Myc forms one H-bond with the hydroxyl group present on the compound's terminal benzene. The glutamic acid present at position 956 forms one H-bond and a salt bridge with another hydroxyl group and NH_2_ group. The Gln at 927 forms one H-bond, each with the two secondary amines of the urea moiety. In addition, the urea moiety oxygen forms one H-bond with the Tyr949 residue of Myc (Fig. 1*D*). For Pol_B_Sul, different amine groups of the compound formed H-bond and salt bridges with the negatively charged residue glutamic acid at positions 957 and 956 (Fig. S2*A*).

### Myc overexpression is associated with cancer and poor patient survival and is expressed in studied cell lines

To assess Myc and cancer's relationship, we investigated the Myc mRNA expression level in various cancer and respective normal samples using the Gene Expression Profiling Interactive Analysis (GEPIA) web-based server. As compared with the healthy samples, the Myc mRNA expression level was significantly higher in all the studied cancer types (Fig. S3). The expression data validate the involvement of Myc overexpression in the pathogenesis of various cancer types. In addition, the Log-rank test, also known as the Mantel–Cox test in the GEPIA webserver, was used to examine the correlation between patient survival and Myc expression level. The 951 high and 1901 low Myc expression samples' analysis revealed an inverse correlation between the Myc mRNA expression level and the patient's overall survival (Fig. S4). The overall survival plot highlighted the prognosis significance of Myc in human cancer. The data mining suggested the overexpression of Myc mRNA in the tumor samples and is related to the prognosis of cancers. A thorough characterization of Myc in the three studied cell lines, that is, D341, HL-60, and HT-29, was done *via* Western blot, quantitative PCR (qPCR), and flow cytometry. All three cell lines showed Myc's endogenous expression at the mRNA and protein level, as depicted from qPCR and Western blot data (Fig. S5, *B* and *C*). The flow cytometry data also supported the same, where a large population of all three cell lines was Myc positive (Fig. S5*A*). Hence, these three cell lines can be used as an *in vitro* model system for assessing the cellular performance of novel compounds having the potential to inhibit Myc–MAX dimerization.

### L755507 is a potent molecule that reduces the growth and restricts cell migration and colony-formation of Myc-expressing cell lines

**Figure 2:**
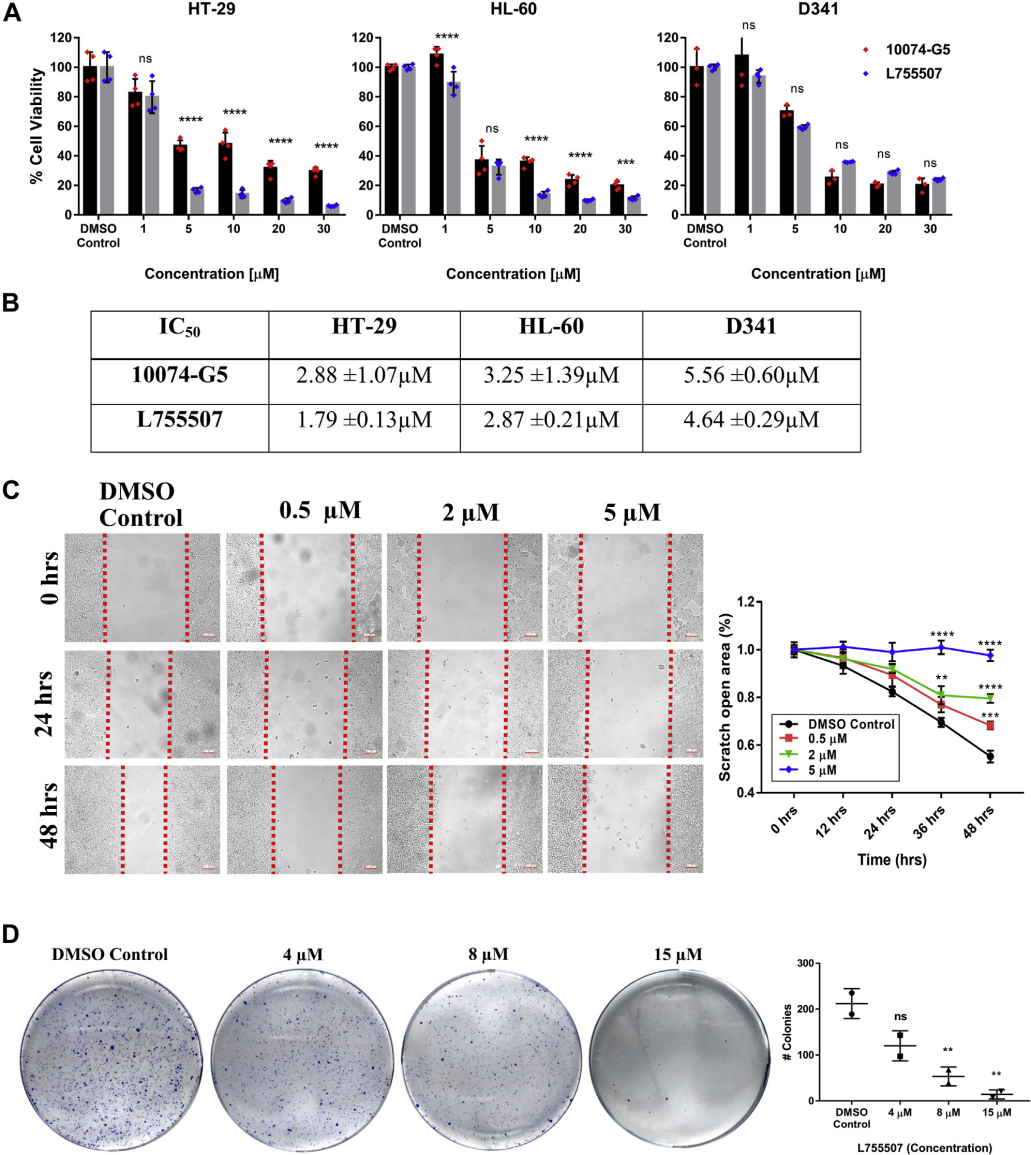
**L755507 showed the anticancer property and restricts the migration and clonal propagation of Myc-expressing cell lines.***A*, anticancer profile of 10074-G5 and L755507 on the three cell lines. *Bars* represent the mean ± SEM of four independent experiments (*p*-value given for 10074-G5 *versus* L755507 for respective concentration. ∗∗∗*p* < 0.001 and ∗∗∗∗*p* < 0.0001). *B*, the obtained IC_50_ values of L755507 along with reported Myc inhibitor, 10074-G5. Values represent the mean ± SEM of four independent experiments. *C*, scratch assay showing restriction in HT-29 cell migration when treated for different concentrations of L755507 for the indicated time (the scale bar represents 100 μm). Data points represent the mean ± SD of three experimental replicates (*p*-value *versus* DMSO control. ∗∗*p* < 0.01, ∗∗∗*p* < 0.001, ∗∗∗∗*p* < 0.0001). *D*, clonogenic assay showing a reduction in the number and size of D341 cell colonies after treatment with different concentrations of L755507 for 10 days. DMSO, dimethyl sulfoxide; ns, nonsignificant.

The structure-based virtual screening potentiated two molecules, that is, L755507 and Pol_B_Sul, with commendable binding affinity and binding energy and was further tested for their cellular potency. For cell-killing potential, both the compounds and 10074-G5 (reported Myc inhibitor) were treated against the three cell lines. After 48 h of treatment, among the two compounds, L755507 showed appreciable cytotoxicity against all three cell lines in a dose-dependent manner (Fig. 2*A*). The IC_50_ value of L755507 was found to be in the lower micromolar range, that is, 1.79 ± 0.13, 2.87 ± 0.13, and 4.64 ± 0.13 μM for HT-29, HL-60, and D341 cells, respectively (Fig. 2*B*). The reported Myc inhibitor, that is, 10074-G5, also showed cell inhibition in a dose-dependent manner, but interestingly, the obtained IC_50_ value was found to be higher than L755507 for the studied cell lines. On the other hand, the second tested compound, Pol_B_Sul, also displayed dose-dependent cytotoxicity but less potency against all three cell lines (Fig. S7), with IC_50_ values on the higher side. In addition, the anticancer activity of L755507 was also studied on low Myc–expressing cell lines (LN-18 and U-87MG) (Fig. S6*A*). From Fig. S6*B*, it can be seen that L755507 showed less cell-killing potential toward the cells with low endogenous expression of Myc, which is evident from its high IC_50_ value (Fig. S6*C*). Based on appreciable cell-killing potential, L755507 was chosen for further cell migration and colony formation assay. The inhibitory potential of L755507 on the mobility of HT-29 cells was studied using scratch/migration assay with different incubation times and concentrations. Analysis of the cell migration to the clear zone showed that L755507 suppresses the migration of HT-29 cells in a dose-dependent manner (Fig. 2*C*). Even after 36 h, treatment with 2 μM and 5 μM of L755507 resulted in a 37.4 and 98.4% reduction, respectively, in cell migration than control cells. Similarly, the long-term colony formation assay showed that L755507 reduces both the size and number of cell colonies in a dose-dependent manner (Fig. 2*D*). After 10 days of incubation, treatment with 8 μM and 15 μM of L755507 displayed a reduction of 75 and 93%, respectively, in cell colonies formed from D341 cells.

### L755507 induces apoptosis and S-phase cell arrest

**Figure 3:**
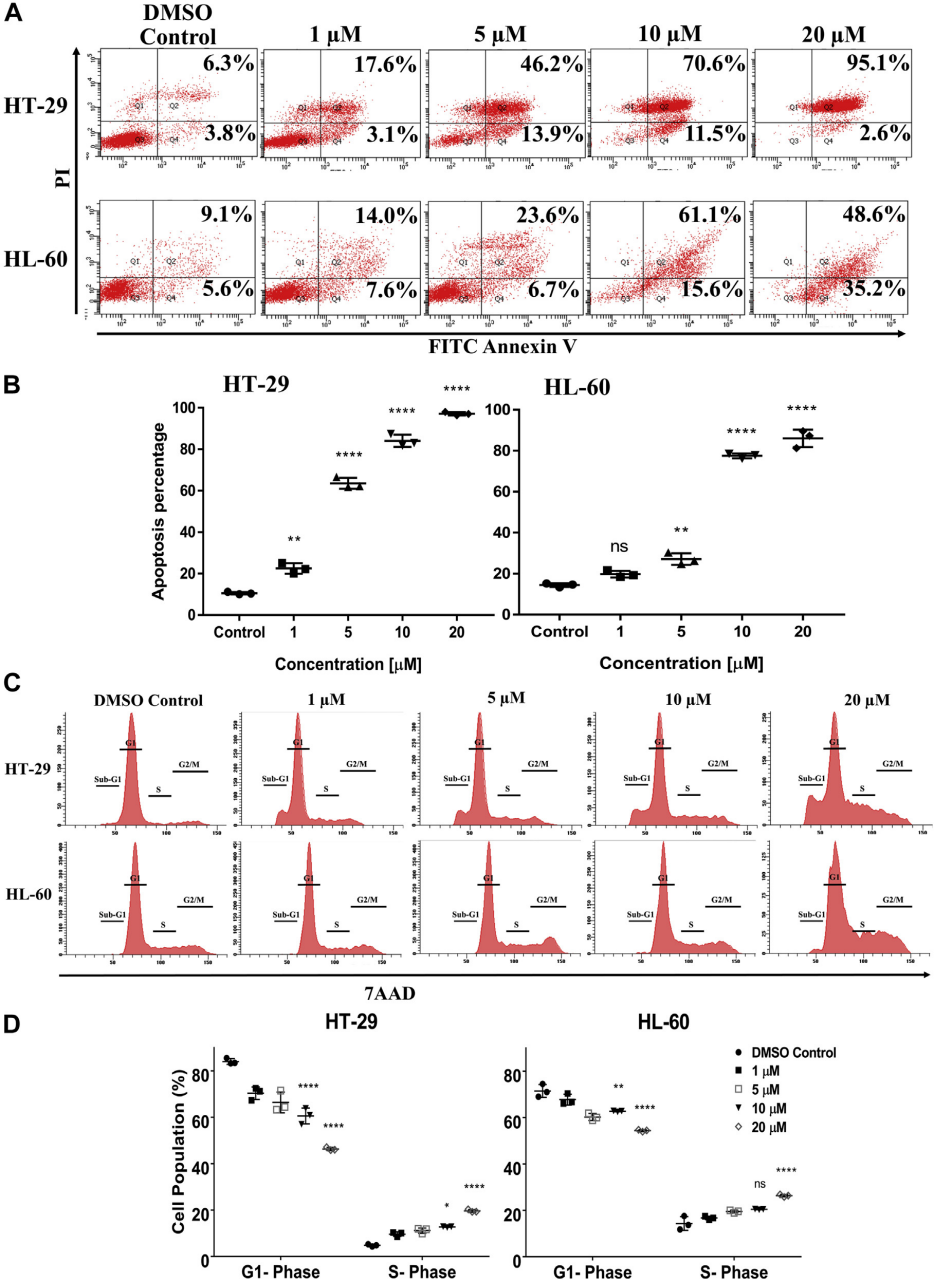
**L755507 induces apoptosis in HT-29 and HL-60 cells by arresting the cells in the S-phase.***A*–*B*, L755507 treatment resulted in apoptosis of both the cell lines in a dose-dependent manner after 36 h. *Plots* represent the mean ± SD of three experimental replicates (*p*-value *versus* DMSO control. ∗∗*p* < 0.01, ∗∗∗∗*p* < 0.0001). *C*, L755507 treatment increases the population of HT-29 and HL-60 cells in the S-phase of the cell cycle in a dose-dependent manner after 36 h. *D*, quantitative representation of percent cell population in a different phase of the cell cycle after treatment with indicated concentrations of L755507. *Plots* represent the mean ± SD of three experimental replicates (*p*-value *versus* DMSO control. ∗*p* < 0.05, ∗∗*p* < 0.01, ∗∗∗∗*p* < 0.0001). DMSO, dimethyl sulfoxide; ns, nonsignificant.

To comprehend the cell death mode, we quantified the cell surface exposure of phosphatidylserine and membrane permeability *via* Alexa Fluor 488-Annexin V/PI molecular probing. After treating HT-29 and HL-60 cells with different concentrations of L755507, the cells were probed with Annexin V/PI and further analyzed in the flow cytometer. As shown in Figure 3*A*, L755507 induces apoptosis in both the studied cell line, which is evident from the increasing proportion of apoptotic cells with increasing L755507 concentration. Interestingly, 97.7% for HT-29 and 83.8% for HL-60 of the total cell population were found to undergo apoptosis when treated with 20 μM of L755507. Deregulation in the cell cycle leads to cancer onset and progression. To study whether the apoptotic cell death caused by L755507 was induced by cell cycle arrest, the HT-29 and HL-60 cells were treated with varying concentrations of L755507. The 7-aminoactinomycin D staining was performed, and the amount of DNA was quantified using flow cytometry. The cell cycle histograms of both the cell lines (Fig. 3, *B* and *C*) showed that the L755507 treatment significantly increases the S-phase cell population with a concomitant decrease in the G1 population in a dose-dependent manner. In addition, an increase in the sub-G1 population demonstrated an increase in apoptosis after L755507 treatment.

### L755507 treatment decreases the expression of Myc target genes *via* abrogating the Myc–MAX dimer

**Figure 4:**
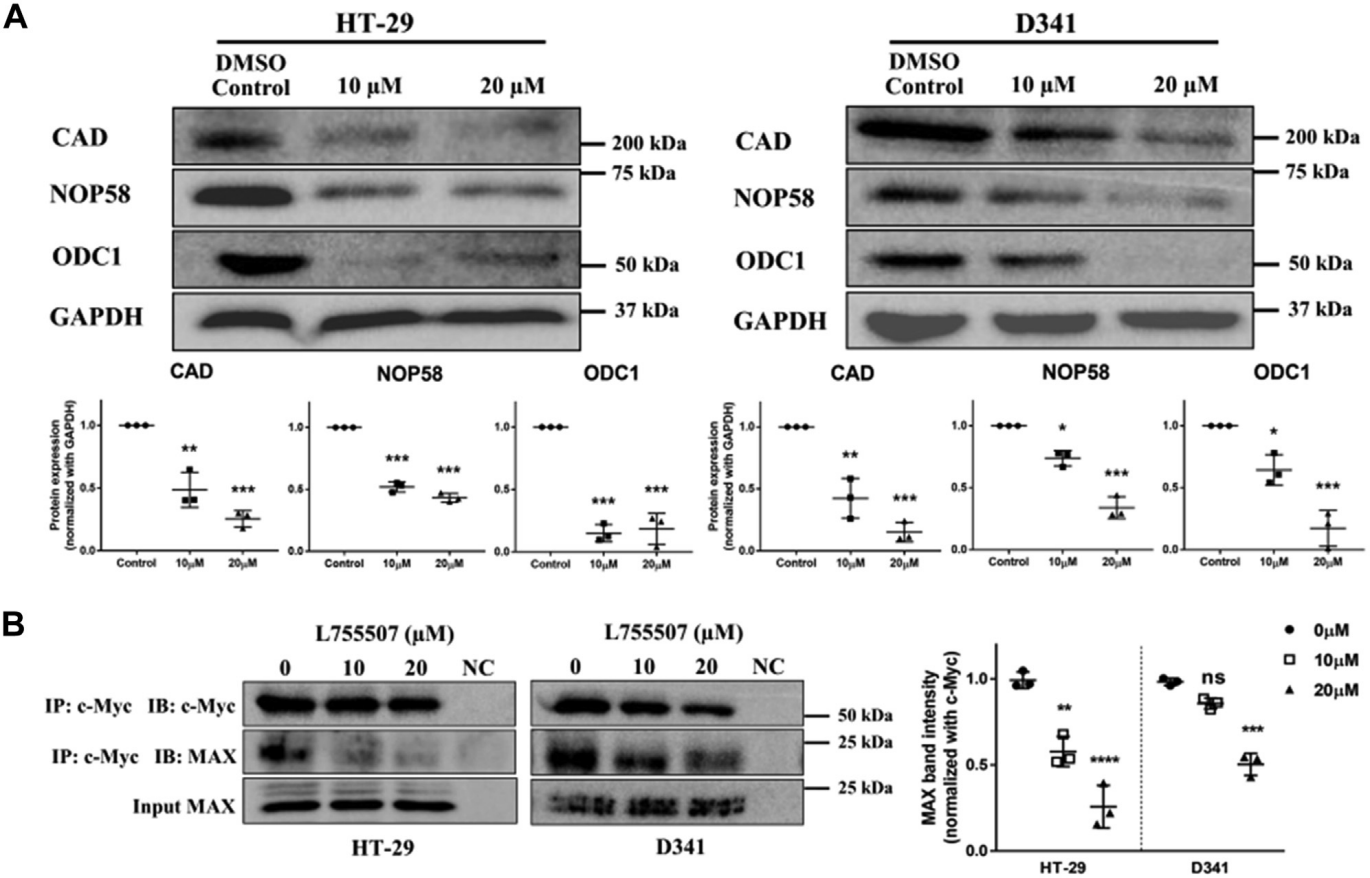
**L755507 treatment leads to repression of Myc target genes by abrogating the Myc–MAX heterodimer.***A*, translational repression of c-Myc target genes induced by L755507. *Plots* represent the mean ± SD of three independent experiments (*p*-value *versus* untreated. ∗*p* < 0.05, ∗∗*p* < 0.01, ∗∗∗*p* < 0.001). *B*, abrogation of Myc–MAX heterodimerization by L755507 in the coimmunoprecipitation assay. *Plots* represent the mean ± SD of three independent experiments (*p*-value *versus* untreated. ∗∗*p* < 0.01, ∗∗∗*p* < 0.001, ∗∗∗∗*p* < 0.0001). ns, nonsignificant.

To further investigate the involvement of Myc inhibition resulting in cancer cell death, we checked the expression of Myc target genes (CAD, ODC1, NOP58, and NOP56 (31, 32)) both transcriptionally and translationally after the treatment of L755507. After treating the three cell lines with increasing concentrations of L755507, a significant decrease in Myc target gene mRNA level was observed compared with untreated cells (Fig. S8). Furthermore, the expression of Myc target genes (CAD, NOP58, and ODC1) after the treatment with L755507 was assessed by Western blot. Similar to the transcriptional data, it was found that the treatment with L755507 leads to the dose-dependent translational repression of all the target genes (Fig. 4*A*). In addition, the effect of 100074-G5 on the expression of Myc target genes was also accessed *via* qPCR and Western blot (Figs. S9 and S10). It was found that even at the higher tested concentration (up to 40 μM; greater than the concentrations used for L755507), 10074-G5 treatment did not display much decrease in the expression of Myc target genes as compared with the L755507. Thus, the qPCR and Western blot studies indicated that L755507 could inhibit Myc's function, and therefore, repression in Myc target genes was observed both at mRNA level and protein level. To comprehend whether the loss of Myc function is due to the inhibition of Myc–MAX dimerization, we performed coimmunoprecipitation experiments. The HT-29 and D341 cells were treated with two different concentrations of L755507, and the Myc immunoprecipitation was carried out from the cell lysate. Immunoblotting with Myc and MAX (Fig. 4*B*) showed that L755507 promotes the dissociation of Myc–MAX dimerization in a dose-dependent manner. For both the studied cell lines, that is, HT-29 and D341, a significant decrease in bounded MAX was observed, whereas no changes were observed in Myc and MAX's overall level when taken as the input control. The data proved that L755507 inhibits the Myc regulatory function by abrogating the association of Myc with MAX.

### Biophysical studies demonstrated the binding of L755507 to Myc

**Figure 5:**
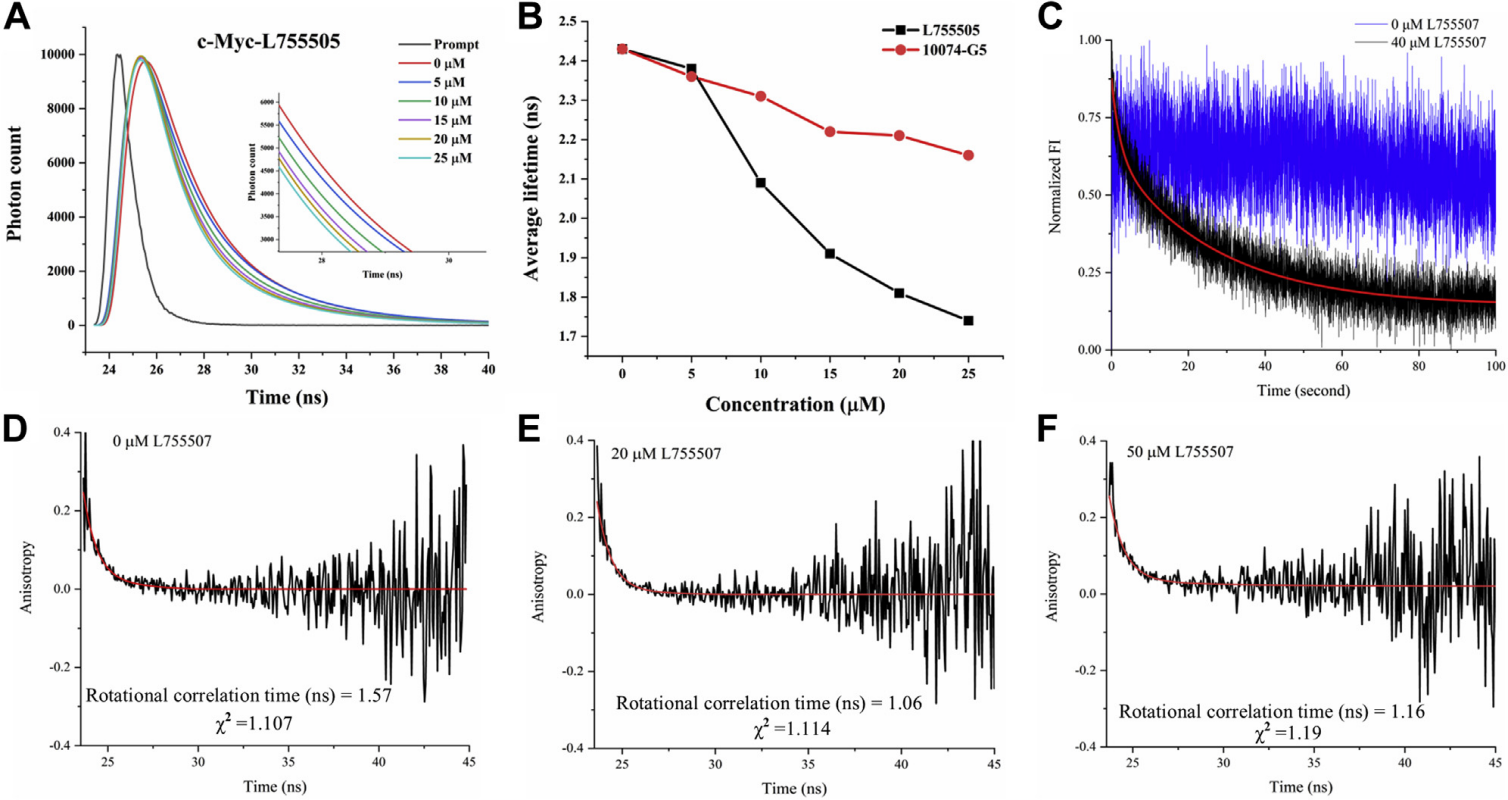
**Effect of L755507 upon interaction with Myc**_**910–960**_**peptide probed *via* Trp fluorescence.***A*, the graph represents the fluorescence lifetime decay measurement of Myc with different concentrations of the ligand L755507. *B*, decrease in average fluorescence lifetime in the presence of L755507 and 10074-G5. *C*, time-resolved fluorescence intensity trace of Myc_910–960_ peptide with 40 μM L755507 obtained by stopped-flow methods (Myc only: *blue* trace; Myc-L755507: *black* trace). The *solid red line* represents the fitting of the curve by the biexponential decay function. Panels *D*–*F* represent the time-resolved anisotropy decay measurement of Myc with 20 μM and 50 μM of ligand L755507. The *solid red line* represents the fitting of the decay by double exponential decay function.

The binding profile of L755507 with Myc_910–960_ peptide was studied using isothermal titration calorimetry (ITC). L755507 showed a high binding affinity toward the Myc peptide, which is apparent from the high association constant (K_A_) value, that is, K_A1_ = 115 ± 5.76 × 10^4^ M^−1^ (Fig. S11). To further gain insight into change in Myc conformation due to L755507 binding, we took advantage of tryptophan (Trp) residue incorporated in the synthetic Myc_910–960_ peptide in place of tyrosine. It is a fact that the Trp fluorescence and corresponding lifetime are affected by the surrounding environment (33). Figure 5*A* shows the fluorescence lifetime decay curve in the presence of L755507, fitted with two exponential decay function. The average lifetime of Myc peptide was 2.43 ns (χ^2^ =0.79) in isolation, and it decreased with the addition of L755507 (Fig. 5*A*). At 25 μM of compound concentration, the fluorescence lifetime declined to 1.74 ns (χ^2^ =0.70) (Fig. 5*B*). The presence of 10074-G5 also resulted in the drop of Myc fluorescence lifetime but not prominent as compared with L755507 (at 25 μM: 2.16 ns (χ^2^ = 0.88)) (Fig. S12 and Fig. 5*B*). The reduction in fluorescence lifetime in the presence of L755507 suggests the interaction of the compound to the Myc peptide, which thereby induces conformational change. We further investigated Myc's conformational dynamics after mixing L755507 (40 μM), using stopped-flow binding kinetics. It is seen that the exponential fluorescence intensity (Trp fluorescence) decays with time (Fig. 5*C*). The decay suggests that the presence of L755507 induces a conformational change that relaxes with time till 100 ns. This decay curve was utilized to determine the rate constants, k_1_ (0.456 ± 0.0181 s^−1^) at the initial stage and k_2_ (0.037 ± 0.00039 s^−1^) at the later stage, demonstrating fast and slow changes in conformational states, respectively. The result obtained from the stopped-flow binding kinetics is in line with fluorescence lifetime measurement, where both are suggesting possible binding/interaction of the L755507 to the Myc peptide. The time-resolved anisotropy decay measurement was used to gain further insight into the dynamics. Figure 5, *D*–*F* represents the time-resolved anisotropy decay of Myc peptide in the presence of L755507. The anisotropy decay curve was fitted with a biexponential decay function, and the curve can be expressed as Equation 1 described in the Experimental procedures section. The average rotational correlation time (t_r_) is 1.57 ns (χ^2^ =1.11) and 1.16 ns (χ^2^ =1.19) in the absence and presence of L755507. Reduced t_r_ may indicate binding of the L755507 to the Myc peptide leads to compaction of Myc global conformation. Overall, the changes in the average lifetime and rotational correlation time measured by lifetime decay and anisotropy decay, respectively, may suggest a possible interaction/binding of L755507 with Myc that influences the global conformational dynamics in Myc.

### Molecular dynamics simulations showed a stable interaction of L755507 with Myc

**Figure 6:**
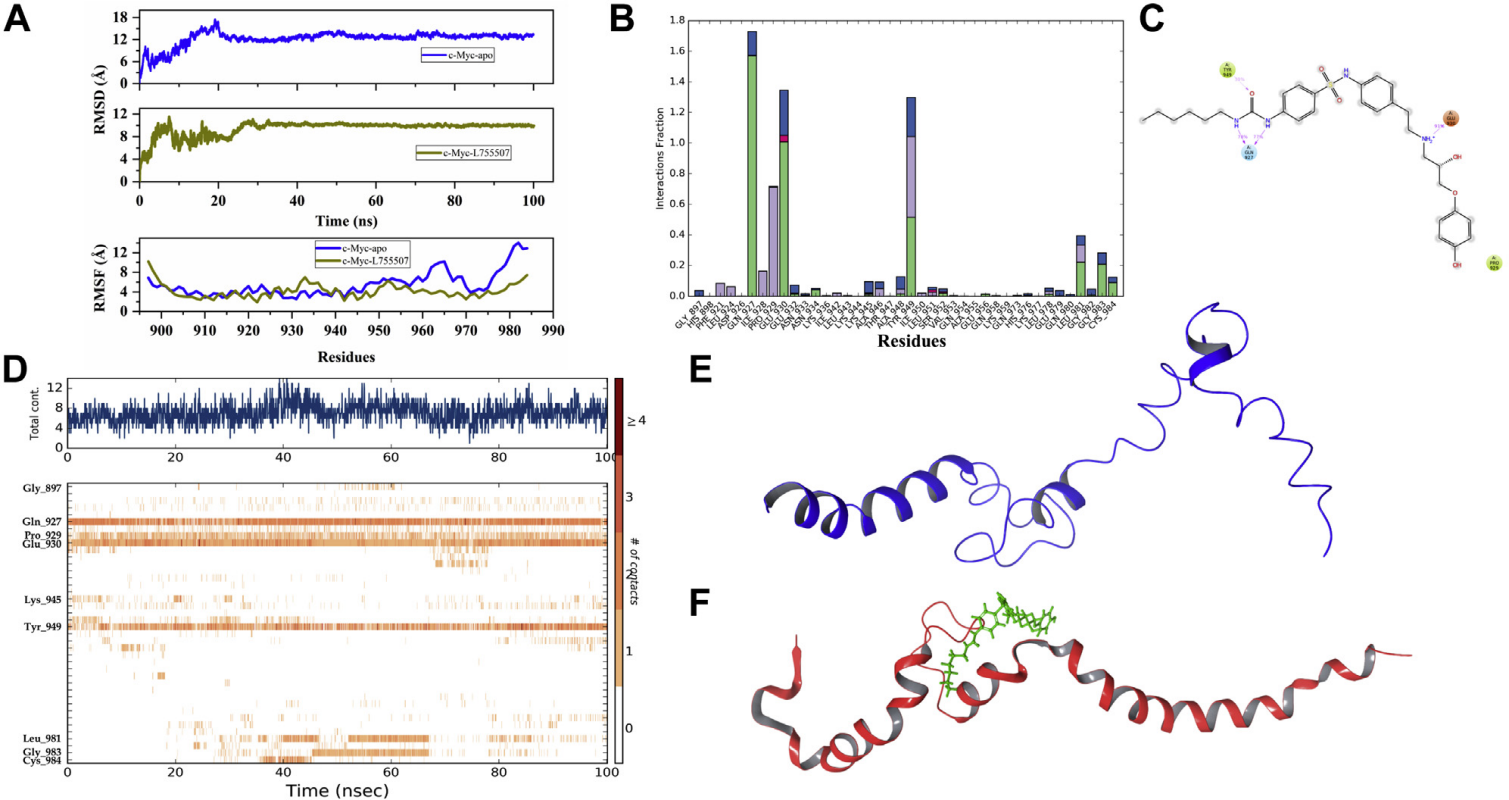
**MD simulation analysis showed a stable Myc/L755507 interaction, and the compound binding stabilizes the helix–loop–helix conformation of the Myc structure.***A*, RMSD of the C-a atoms of unbound Myc (*blue*) and Myc bound to L755507 (*olive*), and root mean square fluctuation (RMSF) in C-a atoms of unbound Myc (*blue*) and Myc bound to L755507 (*olive*). *B*, histogram representation of various interactions formed by residues of Myc with L755507 (values more than 1 show multiple interactions). The color in the bars, that is, *green*, *purple*, *red*, and *blue*, represents hydrogen bonds and hydrophobic, ionic, and water bridge interactions, respectively. *C*, interaction occupancy of residues with compound throughout the simulation period. *D*, timeline depiction of the total number of specific contacts Myc made with L755507 throughout the simulation period. *E* and *F*, the extracted last frame (100 ns) of the simulation trajectory of the Myc-apo (*E*) and Myc/L755507 complex (*F*).

To further validate the Myc/L755507 complex's stability, molecular dynamics (MD) simulations were carried out using the Desmond simulation package. The binding of L755507 with Myc was found to be stable at the scale of 100 ns with strong and intact interactions (Movie S1). The RMSD plot of C-α atoms in the complex has experienced fluctuation initially until 20 ns but stabilized afterward (Fig. 6*A*). The initial fluctuations have caused an increase in RMSD values from 4 to 9 Å for Myc/L755507 complex, which were 4 to 17 Å for Myc's apo conformation. These deviations in Myc have been restricted mostly upon binding with active inhibitor L755507. In root mean square fluctuations (RMSFs), overall fluctuations in apo Myc protein have occurred between 2 and 10 Å. The highest fluctuations are observed in the leucine zipper motif of Myc. Upon binding with L755507, RMSF fluctuations get stabilized in the range of 2 to 7 Å, which is lesser than the unbound form of Myc (Fig. 6*A*). On the other hand, Myc/Pol_B_Sul complex showed higher variation in RMSD ranging from 5 to 20 Å, which was higher than the above two cases (apo-Myc and Myc/L755507) (Fig. S13). Also, the RMSF value of Pol_B_Sul bound Myc structure varies between 4 and 15 Å. Furthermore, MD simulation showed that L755507 formed multiple stable noncovalent interactions with the Myc residues, and the interactions with residues Gln927, Glu930, and Tyr949 remained intact throughout the simulation (Fig. 6, *B*–*D*). Moreover, for apo-Myc, 100 ns of simulation resulted in the loss of helicity in the helix-2 region, which was found to be intact in the L755507-bound Myc structure (Fig. 6, *E* and *F*).

## Discussion

Perturbed protein–protein interaction (PPI) has been responsible for various diseases, including cancer. Myc–MAX interaction is among them, which binds to the E-box sequence at the target gene promoter and is responsible for the oncogenic phenotype (6, 7). PPIs are considered a challenging druggable target, but several small molecules have entered the clinical trials as successful PPI inhibitors (34, 35), including candidates for cancer therapeutics (36, 37). During the last few decades, several attempts have been made to disrupt the dimerization between Myc and MAX, but with little success, the clinical translation of those reported compounds remained impractical (38, 39, 40). Challenges in targeting the Myc–MAX dimer arise because of the highly disordered nature and lack of well-defined binding pockets on Myc's flat interface (11, 39). The bHLH domain of Myc plays a crucial role in maintaining the Myc–MAX dimerization and their subsequent binding to the E-box sequence (19). In the present study, we considered all these challenges by identifying a potential druggable site on the bHLH domain and used that site to discover a bioactive molecule inhibitor that successfully disrupts the Myc–MAX heterodimerization.

A druggable site is characterized by its reasonable size, high solvent exposure, and more hydrophobic and less hydrophilic character (27). Using the Sitemap module's robust algorithm, we chose site 3 to screen bioactive molecules as it justifies all the characteristics of a druggable site (Fig. 1*A*). Structure-based drug design is a distinctive, time-saving, and economical approach widely used to discover new inhibitor molecules. By using virtual screening together with ligand–protein binding energy, we discovered two molecules, that is, L755507 and Pol_B_Sul, with notable binding affinity far superior to the known Myc inhibitors reported to bind to the residues shaped in site 3 (28, 29) (Fig. 1*D*, Fig. S2*A* and Table S4). Among these two bioactive molecules, L755507 interacted with four different Myc residues (Gln927, Glu930, Tyr949, and Glu956). The functional relevance of these interacting residues can be realized by their involvement in interaction with MAX and reported Myc inhibitors. For instance, the Gln927 of Myc residue interacts with the Gln251 of the partner, MAX (41). Moreover, all these four residues, Gln927, Glu930, Tyr949, and Glu956, were previously reported to interact with PKUMDL-YC-1205 (30), PKUMDL-YC-1204 (30), and 10058-F4 (12) Myc inhibitors.

Myc amplification has been reported to various cancers, and its aberrant expression has been reported in various cancers displaying the worst prognosis (6). To assess Myc and cancer's relationship, we investigated the Myc mRNA expression level in various cancer and respective normal samples. Through data mining of The Cancer Genome Atlas and Genotype-Tissue Expression databases using GEPIA, we find that Myc's expression was significantly high in various cancer. Its overexpression is inversely linked to the overall patient survival, highlighting the prognostic role and therapeutic potential of the Myc in cancer. The 3-(4,5-Dimethylthiazol-2-yl)-2,5-diphenyltetrazolium bromide–based cell-cytotoxicity experiments on three Myc-expressing cell lines were performed to study the *in vitro* potency of the two hit molecules in which L755507 stands out as a favorite, with an appreciable cytotoxicity potential and IC_50_ values much lesser than the known Myc inhibitor, 10074-G5 (Fig. 2, *A* and *B*). On the contrary, L755507 showed less cell-killing potential and high IC_50_ values against cells with low endogenous Myc expression (Fig. S6). This suggests the specificity of L755507 toward Myc, leading to cancer cell death. Myc expression is associated with metastasis and is parameterized by the migration capability of the cancer cells. In addition, colony formation, an inherent property of a few single cells to undergo clonal expansion and form a large colony, is also attributed to Myc overexpression (42, 43). Our study found that L755507 efficiently restricts the migration and colony formation property of Myc-expressing cells (Fig. 2, *C* and *D*). We further studied the binding affinity of L755507 analogs toward Myc. Nine substructures of L755507 were downloaded from the PubChem database and docked against the identified site 3 on Myc. It was found that none of the analogs showed superior binding affinity toward site-3 of Myc than L755507 (Table S5). The data suggest that the functional group of L755507 contributes significantly to its binding with the Myc.

Till now, no studies have reported the *in vitro* anticancer activity of L755507. L755507, with molecular weight 584.73 g/mol, is characterized as an extremely potent and selective β3 adrenergic receptor agonist with excellent selectivity, >440 times over other subtypes β1 and β2 (44). A recent study reports that L755507 enhances the CRISPR-mediated homology-directed repair efficiency by nine folds (45). Lipinski's rule of five describes the molecular property of any candidate molecule's pharmacokinetics in the human system. The rule of five comprises specific chemical and physical properties like H-bond donors and acceptors, molecular weight, and the octanol-water partition coefficient. An active drug candidate should not violate more than one criterion of the rule of 5. The drug likeness of L755507 was envisaged using the QikProp (Schrödinger) and OSIRIS DataWarrior (46) programs widely used by the medicinal chemist to define druggability. As seen from Table S6, L755507 satisfies all the pharmacokinetic properties and falls very well in the recommended range defined in the two used programs.

Myc regulates the expression of cell cycle inhibitors and genes (cyclins and CDKs) involved in cell cycle control, and therefore, its inhibition leads to cell cycle arrest. It is reported that Myc expression is linked to an accelerated S-phase, which is marked by replication-associated DNA damage (47), and therefore, its inhibition results in a prolonged S-phase (48). In our study, L755507 treatment resulted in an increased S-phase population with a collateral decrease in the G1 phase cell population (Fig. 3*B*). Our result corroborated with previous reports by Yu *et al.*, (30) where they reported an increase in the S-phase population because of the inhibition of Myc functionality by targeting Myc–MAX. Furthermore, the cell cycle arrest after the treatment with L755507 leads to apoptotic cell death, which was evident from a large proportion of cells undergoing apoptosis when probed with Annexin V/PI.

The onco-dimer formed between Myc and MAX regulates several genes' expression and accounts for the oncogenic phenotype. To confirm the involvement of Myc in L755507-mediate apoptotic cell death, we studied the change in expression of Myc target genes (CAD, ODC1, NOP56, and NOP58 (31, 32)) both at mRNA level and protein level. Our data showed that at both transcriptional and translation levels, the expression of all the studied target genes decreases significantly with increasing concentration of L755507 (Fig. S8 and Fig. 4*A*). Interestingly, the L755507 treatment resulted in a far more decrease in the expression of Myc target genes than the known Myc inhibitor, 10074-G5, used at higher concentrations (Figs. S9 and S10). Furthermore, the Myc immunoprecipitation was carried out to determine the potential of L755507 to abrogate the Myc–MAX heterodimer. Immunoblotting with Myc and MAX (Fig. 4*B*) showed that L755507 promotes the dissociation of Myc–MAX dimerization, which was evident from the significant decrease in bounded MAX, whereas no changes were observed in the overall level of Myc and input control, MAX. Our data suggest that L755507 does not affect the stability and further ubiquitination of Myc. However, Myc destabilization, followed by ubiquitination, has been previously reported while targeting Myc–MAX dimerization (32, 49). Thus, our data indicate that L755507 abrogates the heterodimerization, leading to Myc's functional inhibition, which leads to cell cycle–mediated apoptotic cell death.

Biophysical experiments, including spectroscopic techniques, have been widely used to study small molecules' interaction with the target protein. Therefore, using the spectroscopic techniques such as fluorescence lifetime measurement, anisotropy measurement, and stopped-flow binding kinetics and taking advantage of the single Trp residue incorporated in the synthetic Myc_910–960_ peptide, which acted as an intrinsic fluorescent probe, we monitored the change in Myc confirmation in the presence of L75507. A decrease in fluorescence lifetime and average rotational time was observed with the gradual increase of L755507 that indicates a change in the conformational dynamics of Myc in the presence of the compound (Fig. 5, *A*, *B* and *D*–*F*). Furthermore, the stopped-flow binding kinetics also demonstrated the time-dependent conformational relaxation of the Myc peptide with L755507. Here, we found that the fluorescence intensity decreases exponentially as the function of time (Fig. 5*C*) suggests that the binding of L755507 influences conformational dynamics in Myc. Results of all three spectroscopic techniques are in a concordance and suggest a change in Myc conformation in the presence of the L755507 that mirrored the binding of L755507 to the Myc peptide.

In the unbound state, the C-terminal region constituting Myc's bHLH-LZ domain is highly disordered and undergoes ordered transformation *via* adopting a stable helical conformation after dimerizing with the partner protein MAX (20, 21). This flexible conformation of Myc is essential for its dimerization with MAX. This study has shown that L755507 can efficiently disrupt heterodimerization, and, therefore, to examine the stability of Myc/L755507 complex and to understand the Myc's structural conformation in complex, we used 100 ns of extensive computational simulations using the Desmond tool. As per simulation parameters such as RMSD and RMSF, a stable binding and an overall less fluctuation of the Myc/L755507 complex was observed compared with the unbound Myc (Fig. 6*A* and Movie S1). Moreover, it was observed that the interaction of residues, Gln927, Glu930, and Tyr949 of Myc with L755507 remained intact throughout the simulation period (Fig. 6*C*). On the other side, the second hit, Pol_B_Sul, selected from visual inspection, showed higher deviations than unbound Myc (Figs. S13 and S14). In addition, we also observed that after an extended period of simulation, the apo-Myc lost its helicity to the disordered structure, whereas the binding of L755507 resulted in a stable bHLH conformation of the Myc structure (Fig. 6, *E* and *F*). Therefore, it can be speculated that L755507 binding to the region induces Myc's overall structural stability, thereby preventing the dimerization with partner MAX. Our computational data correlate well with the biophysical studies and suggest that L755507 binds explicitly to Myc's bHLH domain, thereby inhibiting the onco-dimerization leading to functional repression of Myc.

Various efforts have been made to discover small molecules that disrupt the dimerization of c-Myc and MAX (6, 19). Most of these inhibitors display low potency in the *in vitro* setup with an IC_50_ value in the high micromolar range (11, 12, 50). Among all these reported inhibitors, only a few such as 10058-F4, 10074-A4, 10074-G5, and KJ-Pyr-9 have been studied using biophysical experiments for their binding with the c-Myc peptide (11, 28, 51). The promising advantage that L755507 upholds from the reported inhibitors is its low micromolar IC_50_ values against Myc-expressing cell lines, which is translated because of its potential to inhibit the Myc's functionality by efficiently abrogating the heterodimer formation. In addition, the biophysical studies reflect its high binding potential against the Myc peptide, leading to conformational changes.

The study collectively reported the implication of *in silico* drug discovery approach to target Myc's flat interface that lacks selective binding sites for small-molecule inhibitors. Combining the results of rigorous molecular docking and binding energy analysis, we identified top hit molecules having the potential to disrupt the Myc–MAX interaction. L755507, a β3 adrenergic receptor agonist, was reported as a lead molecule that efficiently restricts the growth of Myc-expressing cells *via* selectively disrupting the Myc–MAX dimerization. Comprehensively, the study provides a conceptual basis of L755507 direct binding and forming a stable complex with Myc, which ultimately results in the modulation of Myc transcriptional regulation property. Our study showed that L755507 inherits an exceptional Myc–MAX dimer inhibiting property. Its further exploration in the preclinical setup for efficacy and pharmacokinetics behavior will render it a promising Myc-targeted antineoplastic drug. In addition, L755507 also serves as a new lead molecule that can be further functionally improved to achieve a highly effective binding while upholding biological efficacy as a therapeutic agent.

## Experimental procedures

### Cell culture

The HL-60 (human female, promyelocytic leukemia) and HT-29 (human female, colorectal adenocarcinoma) cells were procured from the National Centre for Cell Science, Pune, India. The D341 (human male, medulloblastoma) cell line was procured from the American Type Culture Collection (Manassas). HL-60 and HT-29 cells were maintained in RPMI 1640 (Thermo Fisher Scientific) and Dulbecco's modified Eagle's medium (HyClone, GE), respectively, supplemented with 10% fetal bovine serum (Sigma Aldrich), 1% Antibiotic-Antimycotic (Thermo Fisher Scientific), and 1% sodium pyruvate (Thermo Fisher Scientific). The D341 cells were maintained in Eagle's Minimum Essential Medium (BD Biosciences) supplemented with 20% fetal bovine serum and 1% Penicillin-Streptomycin (Thermo Fisher Scientific). All the cells were grown in a humid environment at 37 °C with 5% CO_2_.

### Protein structure preparation and druggable site prediction

The crystal structure with high resolution, that is, 1.80 Å of Myc–MAX heterodimer bound to the enhancer sequence, was downloaded from the PDB (PDB ID: 1NKP (26)). By utilizing the Protein Preparation Wizard utility of Schrödinger Release 2018-4: Maestro, Schrödinger, L.L.C., New York, NY, 2018, we prepared the structure of Myc by adding missing hydrogens, determining correct ionization and protonation states and then optimized its hydrogen bond network. After that, the restrained minimization was performed to minimize the hydrogen atoms, to relax the steric clashes and strained bonds using OPLS 2005 forcefield. To identify a potential druggable site on the flat and large interface, only the crystal structure of Myc (chain A, without MAX and DNA) was subjected to the Sitemap program (27) of Schrodinger. With a remarkable prediction of balanced as well as standalone hydrophobic and hydrophilic site points, hydrogen bond donor/acceptors properties, it predicted five active sites. These sites were ranked based on the draggability score, site score, and hydrophobicity/hydrophilicity scores.

### Molecular docking

The bioactive molecules from Selleckchem (Bioactive Compound Library-I and Bioactive Compound Library-II) were selected for screening against the identified site on Myc. According to the provider's information, the safety of several of these compounds has been tested in preclinical and clinical setups. The .sdf format of the two libraries of bioactive compounds consisting of 12,433 compounds was downloaded from Selleckchem online database. These compounds were then prepared using the LigPrep module (52) of Schrodinger. LigPrep generates the 3D conformations with minimized energy, and all other corrected required chemical parameters such as ionization and tautomeric states and stereoisomers of each processed compound in less time. The prepared structures were then subjected to docking against the identified active site in the Myc structure. Schrodinger's Glide (53) performs docking of the compound using an empirical scoring function that utilizes OPLS 2005 force field to estimate the binding affinities and provide potential hits. The Extra precision (XP) of Glide was implemented, which reduces the false-positive binding poses and generates accurate ones. After identifying the active site on the prepared structure for docking, a receptor grid was generated to perform the search of the best hits. The grid center coordinates were 69.22, 74.59, and 37.5 for x, y, and z, respectively. The binding free energy (dG) values were also calculated using the molecular mechanics-generalized born surface area approach, where the difference of energies of the receptor–ligand complex and receptor and ligand separately is accounted along with solvation energy. All the docked compounds were manually screened based on binding energy, docking score, and interactions with the key residues.

### MD simulations

The selected hit compounds against Myc were tested for their binding stability and compared with the unbound conformation of Myc for up to 100-ns simulation time. Desmond simulation package, developed by D. E. Shaw Research, was implemented for this purpose using previously described methods (54, 55). The simulation system was set up using System Builder that places the protein–ligand complex into an orthorhombic box with a distance of 10 Å from each edge. The system was neutralized by adding counterions, and 0.15 M NaCl salt concentration was added for proper electrostatic distribution. Furthermore, the minimization of the simulation system was carried out using the steepest descent and LBFGS method for 2000 iterations, followed by equilibration using the NPT ensemble for 1 ns. Finally, the production run for 100 ns was performed at an average 310K temperature and 1 bar pressure. For maintaining the average temperature and pressure, Berendsen thermostat and barostat were applied. The simulation outcomes were visualized in maestro and analyzed using the Simulation Interaction Diagram and Simulation Event Analysis programs of Desmond.

### Data mining and Myc gene profiling

Compared with normal samples, the Myc mRNA expression level in different cancer samples was analyzed from the GEPIA online database (http://gepia.cancer-pku.cn/). GEPIA is a web-based platform that provides customizable functions based on The Cancer Genome Atlas and the Genotype-Tissue Expression database (56). The “Boxplot” tab was used to investigate the Myc mRNA expression in the three cancer patients compared with normal data. For overall patient survival, the Mantel–Cox test was used for hypothesis testing, including the Cox proportional hazard ratio and the 95% confidence interval.

### Cell viability assay

Cells (5 × 10^3^) of all three cell lines were seeded in a 96-well plated and incubated with different concentrations of 10074-G5 (Cat No. 475957, Merk Millipore), L755507 (Cat No. SML1362, Sigma Aldrich), and Polymyxin B Sulfate (Cat No. P4932, Sigma Aldrich) for 48 h along with all experimental controls. Dimethyl sulfoxide (0.2%) was used as the vehicle control with all cell lines. After 48 h of incubation, 10% 3-(4,5-Dimethylthiazol-2-yl)-2,5-diphenyltetrazolium bromide was added to each well and further incubated for 2.5 h. The formazan crystals were dissolved with dimethyl sulfoxide, and the absorbance was recorded at 570 nm with 650 nm as a reference in the Tecan Infinite M200 PRO plate reader. The data were analyzed in GraphPad Prism 7, and the IC_50_ value was calculated by [inhibitor] *versus* response–Variable slope (four parameters) in GraphPad Prism 7.

### Real-time qPCR

The cells were treated with different concentrations of L755507 and 10074-G5 for 36 h, and the total RNA was isolated using RNA-XPress reagent (HiMedia) following manufacturer's protocol. The RNA was quantified using Qubit 3.0 Fluorometer (Life Technologies), and 1 μg RNA was used for cDNA synthesis using iScript Select cDNA synthesis kit (Bio-Rad), including both oligo(dT)_20_ and random primers in an equal ratio. qPCRs were set up using Hi-SYBr Master Mix (HiMedia), and the reaction was quantified in CFX96 Touch Real-Time PCR Detection System (Bio-Rad). A complete list of primer sequences used during the qPCR is provided in Table S2.

### Western blot

D341 and HT-29 cells were treated with different concentrations of L755505 and 10074-G5 for 36 h. Cell pellets were lysed using RIPA buffer (25 mM Tris·HCl, pH 7.6, 150 mM NaCl, 1% NP-40, 1% sodium deoxycholate, 0.1% SD.S.) along with Halt Protease Inhibitor Cocktail (Thermo Fisher Scientific). The protein concentration was further determined by Pierce BCA Protein Assay Kit (Thermo Fisher Scientific). An equal volume of protein was electrophoresed using SDS-PAGE gels, and the bands were transferred to preactivated polyvinylidene fluoride membranes (Merck Millipore). After blocking with 5% skim milk powder (HiMedia), the blots were probed with primary antibodies at 4 °C overnight. After washing, the blots were incubated with respective horseradish peroxidase–conjugated secondary antibodies for 1 h at room temperature (RT). The blot was then incubated with Clarity Western ECL Substrate and imaged in Amersham Imager 600 (GE), and the band intensity was quantified using ImageJ software. The list of antibodies used in Western blot is provided in Table S1.

### Flow cytometry

Flow cytometry analysis was performed to characterize the Myc expression in the three cell lines. The single-cell suspension of 1 × 10^6^ cells was fixed and permeabilized for 20 min in ice using BD Cytofix/Cytoperm kit (BD Biosciences). The cells were further incubated with FITC-conjugated anti-Myc antibody and FITC-conjugated isotype control on ice for 45 min. The Myc-positive cells were quantified in LSRFortessa cell analyzer (BD Biosciences) by collecting 10,000 events per sample. The data acquisition and analysis were performed using BD FACSDiva software. The list of antibodies used in flow cytometry is provided in Table S1.

### Annexin V/PI apoptosis assay

HT-29 and HL-60 cells were seeded in a 12-well plate at a density of 1 × 10^6^ cells per well and treated with different concentrations of L755507 for 36 h. In addition, negative and single stain controls were used to set the voltage and fluorescence compensation. The harvested cells were washed with PBS and resuspended in Annexin binding buffer. Cells were then further incubated with Annexin V-Alexa Fluor 488 conjugate (Cat No. A13201, Thermo Fisher Scientific) and propidium iodide (Cat No. P3566, Thermo Fisher Scientific) for 20 min at RT. Ten thousand events per sample were collected in an LSRFortessa cell analyzer, and the data acquisition and analysis were achieved using BD FACSDiva software.

### Cell cycle analysis

For cell cycle analysis, 1 × 10^6^ cells of HT-29 and HL-60 were treated with different concentrations of L755507 for 36 h. After harvesting and washing with ice-cold PBS, the cells were fixed with ice-cold 70% ethanol for 3 h at a temperature below 4 °C. After subsequent washing with ice-cold PBS, the cell samples were incubated with 7-aminoactinomycin D (Cat No. A1310, Thermo Fisher Scientific) for 30 min at RT. The cells were quantified in an LSRFortessa cell analyzer with 10,000 events per sample, and the data acquisition and analysis were made using BD FACSDiva software.

### Migration assay

HT-29 cells were seeded in a 24-well plate and allowed to grow till the confluency reaches ≈80%. Once the confluency was attained, perpendicular scratches were made using a 10-μl pipette tip, and the cells were washed with PBS to remove all the debris and suspended cells. After L755505 treatment, the images of scratches were taken at 0, 12, 24, 36, and 48 h using Axio observer 5, Carl Zeiss Fluorescence Microscope. The scratch area was quantified using ImageJ software.

### Colony-forming assay

D341 cells were seeded in a 6-well plate at a density of 1000 cells per well with varying concentrations of L755507. The cells were further incubated for 10 days with the addition of fresh media after 5 days. After 10 days of treatment, the cells were fixed with 4% formaldehyde and stained with 0.2% Coomassie Brilliant Blue for 30 min. The colonies were quantified using ImageJ software.

### Coimmunoprecipitation assay

For coimmunoprecipitation, D341 and HT-29 cells were treated with different concentrations of L755507 for 36 h. After harvesting, the cells were washed with ice-cold PBS, and the pellet was lysed with IP lysis buffer (50 mM Hepes, 300 mM NaCl, 1% Triton X-100, 10 mM MgCl_2_, 15% glycerol, pH 7.9) supplemented with Halt Protease Inhibitor Cocktail. The lysate was cleared by centrifugation at 16,000*g* for 15 min at 4 °C. 50 μg of cellular lysate was incubated with 3 μg of anti-Myc mouse primary antibody at 4 °C overnight. Furthermore, 1 mg of prewashed SureBeads Protein G (Cat No. 161-4023, Bio-Rad) was used to precipitate the Myc–MAX complex by incubating at 4 °C for 4 h. The magnetized Protein G beads along with precipitate were further washed trice with IP wash buffer (25 mM Hepes, 300 mM NaCl, 0.2% Triton X-100, pH 7.9), resuspended in the protein loading buffer, and separated *via* 12% SDS-PAGE. Negative and input Myc control was used for comparison in all sets of experiments. Furthermore, MAX was immunoblotted by anti-MAX rabbit primary antibody, and input Myc was immunoblotted by anti-Myc mouse primary antibody. The list of antibodies used in coimmunoprecipitation is provided in Table S1.

### ITC

ITC measurements were done at 25 °C on a VP-ITC microcalorimeter from MicroCal, Inc (GE, MicroCal) as per previous protocols (57). The first injection was a false one of 2 s with other injections set at 10 s. The sample cell was filled with peptide while the syringe was filled with the ligand of interest. The stirring rate was maintained at 320 rpm. The obtained titration data were analyzed by Origin 8.0 software to estimate thermodynamic parameters such as stoichiometry of binding (n), enthalpy change (ΔH), and association constant (K_A_).

### Fluorescence lifetime measurement

For biophysical validation, Myc_910–960_ peptides were purchased from Thermo Fisher Scientific with 90% purity. The fluorescence lifetime of Trp in Myc peptide was measured using the DeltaFlex TCSPC system (Horiba Scientific). A quartz cuvette of 1 cm × 1 cm and 1 ml volume were used to record the spectra. The excitation monochromator's wavelength was set up at 284 nm, and the emission monochromator at 345 nm. The measurement range was set up to 200 ns with 32 nm of bandpass and peak preset of 10,000 counts. LUDOX was used to correct the instrument response factor at 284-nm wavelength. 7.5 μM Myc peptide sample with L755507 and 10074-G5 was prepared in 50 mM sodium phosphate buffer (pH 7) and incubated for 10 min at 25 °C before taking the measurement. The decay curve was fitted into a biexponential decay function using Data Analysis Software provided by Horiba Scientific.

### Time-resolved anisotropy decay measurement

The anisotropy decay was measured using the DeltaFlex TCSPC system (Horiba Scientific). A quartz cuvette of 1 cm × 1 cm and 1 ml volume were used to record the spectra. The measurement range was set up to 200 ns with 16 nm of bandpass, peak preset of 1000 counts, and repetition rate of 1 MHz. The protein sample (20 μM Myc) with compound L755507 was incubated for half an hour at 25 °C before taking the measurement. LUDOX was used for correcting the instrument response factor at 284-nm wavelength. The anisotropy decay curve was fitted into two exponential decay function using Data Analysis Software provided by Horiba Scientific. The anisotropy decay curve can be expressed by the following equation (33).

(1) $$r\left(t\right)=r_{0}\left[A_{1}exp\;\left(-\;\frac{t}{\theta_{1}}\right)\;+\;A_{2}exp\;\left(-\;\frac{t}{\theta_{2}}\right)\right]$$

Where, *r(t)* is the anisotropy at time *t*, *r*_*0*_ is the intrinsic or initial anisotropy, *A*_1_ and *A*_2_ are the amplitude associated with the rotational correlation time $\theta_{1}$ and $\theta_{2}$, respectively.

### Stopped-flow binding kinetics

The rapid mixing of Myc peptide and compound, L755507, was obtained using SFM 3000 (Bio-Logic Science Instruments). The mixing dead time and total flow rate of the mixture were set to 4.6 ms and 8 ml/s, respectively, for the FC15 sample cuvette. The time-dependent change was monitored by fluorescence emission measurement at 290-nm excitation wavelength using a 320-nm cut-off glass filter. The bandwidth was adjusted to 4 nm. Because the dead volume for the FC15 cuvette is 51 μl, pushing the sample more than three times the dead volume was done for each acquired trace. The final concentration of Myc and L755507 in the mixed sample was 0.4 μM and 40 μM, respectively. The average of three traces of the decay curve was fitted with a double exponential function to obtain the rate constant.

### Statistical analysis

Three technical/experimental replicates were considered for all experiments unless specified in the figure legends. The data are presented as the mean ± SD or SEM, and the number of replicates is given in the figure legends. Differences were analyzed by using the Student's *t* test and ANOVA in GraphPad Prism 7. Results with *p*-value < 0.05 were considered statistically significant.

## Data availability

The data generated and supporting this article's conclusion are included in the main text file and supporting information.

## Supporting information

This article contains supporting information (12, 30).

## Conflict of interest

The authors declare that they have no conflicts of interest with the contents of this article.
