# Supplementary material for: A novel inhibitor L755507 efficiently blocks c-Myc–MAX heterodimerization and induces apoptosis in cancer cells
Source: J Biol Chem. 2021 Jun 23;297(1):100903. doi: 10.1016/j.jbc.2021.100903 (PMC8294579; doi:10.1016/j.jbc.2021.100903)
Supplement: Supplemental Figures S1–S14 and Tables S1–S6 [file mmc1.pdf]

A novel inhibitor L755507 efficiently blocks c-Myc/MAX heterodimerization and induces apoptosis in cancer cells

Ashutosh Singh[1], Ankur Kumar[1], Prateek Kumar[1], Namyashree Nayak[1], Taniya Bhardwaj[1], Rajanish Giri[1], Neha Garg[2]*

[1]School of Basic Sciences and Advanced Materials Research Center, Indian Institute of Technology Mandi, Kamand, Mandi, 175005, Himachal Pradesh, India

[2]Department of Medicinal Chemistry, Institute of Medical Sciences, Banaras Hindu University, Varanasi- 221005, Uttar Pradesh, India

***Corresponding Author**

Neha Garg, Ph.D.

Department of Medicinal Chemistry,
Institute of Medical Sciences, Banaras Hindu University,
Varanasi- 221005, Uttar Pradesh, India
Telephone: +91-9818082907
Email: nehagarg@bhu.ac.in

Running title: L755507 abrogates the dimerization of Myc/MAX.

Figure S1. Dot plot depicting docking score and binding energy of top 178 compounds from Selleckchem Bioactive library (blue squares) and reported Myc/MAX inhibitor from the literature (red squares). The arrow denotes the position of L755507 corresponding to its binding energy and docking score.

Figure S2. High-throughput virtual screening aided in the discovery of hit bioactive compounds against oncogenic Myc. (A) The potential binding poses and respective ligand molecular interactions of second hit molecule, Pol_B_Sul, and reported Myc inhibitors, 10074-G5 (B) 10074-A4 (C) and PKUMDL-YC-1204 (D) with the identified Site-3 residues of Myc.

Figure S3. Gene Expression Profiling Interactive Analysis (GEPIA) was performed to validate Myc mRNA expression in various cancer samples vs. normal samples. Data represented as mean ± SD (*p < 0.05). (Abbreviation: CECS-Cervical squamous cell carcinoma and endocervical adenocarcinoma, CHOL-Cholangiocarcinoma, COAD-Colon adenocarcinoma, DLBC-Lymphoid Neoplasm Diffuse Large B-cell Lymphoma, ESCA- Esophageal carcinoma, GBM- Glioblastoma multiforme, KIRC- Kidney renal clear cell carcinoma, KIRP- Kidney renal papillary cell carcinoma, LGG- Brain Lower Grade Glioma, LUSC- Lung squamous cell carcinoma, PRAD- Prostate adenocarcinoma, READ- Rectum adenocarcinoma, STAD- Stomach adenocarcinoma, and THYM-Thymoma)

Figure S4. Mantel-Cox survival curve represents high Myc expression with reduced overall survival in different cancer types compared with low Myc expression.

Figure S5. Endogenous expression of Myc in three cell lines viz. D341, HL-60, and HT-29 were characterized by (A) flow cytometry, (B) western blot, and (C) real-time quantitative PCR.

Figure S6. Cytotoxicity result of L755507 on low-Myc expressing cell lines. (A) Endogenous expression of Myc on two glioma cell lines, i.e., LN-18 and U-87 MG, was probed with western blot and compared with HT-29. (B) Anticancer profile of L755507 on LN-18 and U-87 MG cells after 48 hrs of treatment. The bar represents the mean ± SEM of three independent experiments. (C) The obtained IC50 values of L755507 against the two low-Myc expressing cell lines. Values represent mean ± SEM of three independent experiments.

Figure S7. Cytotoxicity result of the Pol_B_Sul on three Myc expressing cell lines. (A) Anticancer profile of Pol_B_Sul on three cell lines. The bar represents mean ± SEM of three independent experiments. (B) The dose-response curve used to generate the IC_{50} value of Pol_B_Sul on three studied cell lines. Data points represent mean ± SEM of three independent experiments. (C) The obtained IC50 values of Pol_B_Sul. Values represent mean ± SEM of three independent experiments.

Figure S8. L755507 treatment resulted in the inhibition of Myc transcriptional activity, which is evident from the decreased mRNA level of Myc target genes (CAD, ODC1, NOP58, and NOP56). All three cell lines were treated with the indicated concentration of L755507 for 36 hrs and subjected to qPCR. Bars represent mean ± SD of three experimental replicates (p-value vs DMSO control. *p<0.05, **p<0.01, ***p<0.001, ****p<0.0001, ns = non-significant).

Figure S9. The mRNA expression level of the Myc target gene (CAD, ODC1, NOP58, and NOP56) after treatment with various concentrations of 10074-G5. All three cell lines were treated with the indicated concentration of 10074-G5 for 36 hrs and subjected to qPCR. Bars represent mean ± SD of three experimental replicates (p-value vs DMSO control. *p<0.05, **p<0.01, ***p<0.001, ****p<0.0001, ns = non-significant).

Figure S10. Change in the protein level of c-Myc target genes (CAD, NOP58, and OD1) after treatment with 10074-G5. Bars represent mean ± SD of two independent experiments (p-value vs untreated. *p<0.05, **p<0.01, ***p<0.001).

Figure S11. Isothermal titration calorimetry isotherm for the interaction of L755507 with Myc peptide. The upper panel relates to calorimetric response as successive injections of L755507 into sample cell containing Myc peptide. The bottom panel corresponds to integrated heats of interactions as a function of [L755507-Myc] molar ratio.

Figure S12. The graph represents the fluorescence lifetime decay measurement of Myc with different concentrations of the ligand 10074-G5.

Figure S13. Molecular dynamics simulations analysis of Myc/Pol_B_Sul complex for 100 ns. Root Mean Square Deviation (RMSD) of the Cα atoms of unbound Myc (blue) and Myc bound to Pol_B_Sul (olive), and Root Mean Square Fluctuation (RMSF) in Cα atoms of unbound Myc (blue) and Myc bound to Pol_B_Sul (olive).

Figure S14. (A) Timeline depiction of the total number of specific contacts the Myc made with Pol_B_Sul throughout the simulation period of 100ns. (B) Histogram representation of various interactions formed by residues of Myc with Pol_B_Sul (values more than 1 show multiple interactions) during a course of computational simulations. The color in the bars, i.e., green, purple, red, and blue, represents hydrogen bonds, hydrophobic, ionic, and water bridge interactions. (C) Interaction occupancy of Site-3 residues with Pol_B_Sul throughout the simulation period.

Table S1. Complete detail of antibodies used in different experiments in this study.

Table S2. List of Real-time quantitative PCR primers used in the study.

Table S3. Predicted shallow active sites on the apo structure of Myc from the Myc-MAX complex generated by SiteMap.

Table S4. Docking score and binding energy of hit bioactive compounds and the reported Myc inhibitors bind to the identified site on Myc.

Table S5: List of sub-structures from PubChem database of L755507 with their docking score and binding energy scores against identified Site-3 of Myc.

Table S6: Prediction of pharmacokinetic properties of L755507 using QikProp and OSIRIS DataWarrior programs. a: Estimated number of H-bond donors; b: Estimated number of H-bond acceptors; c: Predicted octanol/water partition coefficient; d: Predicted aqueous solubility; e: Conformation-independent predicted aqueous solubility.

Movie S1 (separate file). 100ns simulation trajectory of Myc/L755507 complex showed a stable binding of compound (Red) with Myc (Blue).

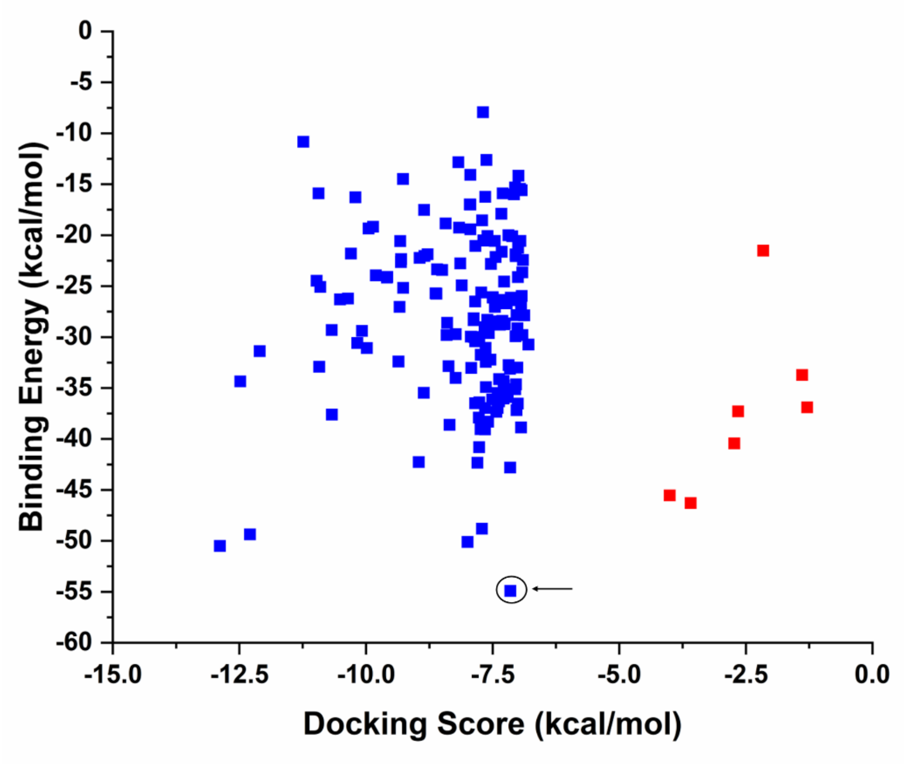

Figure S1. Dot plot depicting docking score and binding energy of top 178 compounds from Selleckchem Bioactive library (blue squares) and reported Myc/MAX inhibitor from the literature (red squares) (1, 2). The arrow denotes the position of L755507 corresponding to its binding energy and docking score.

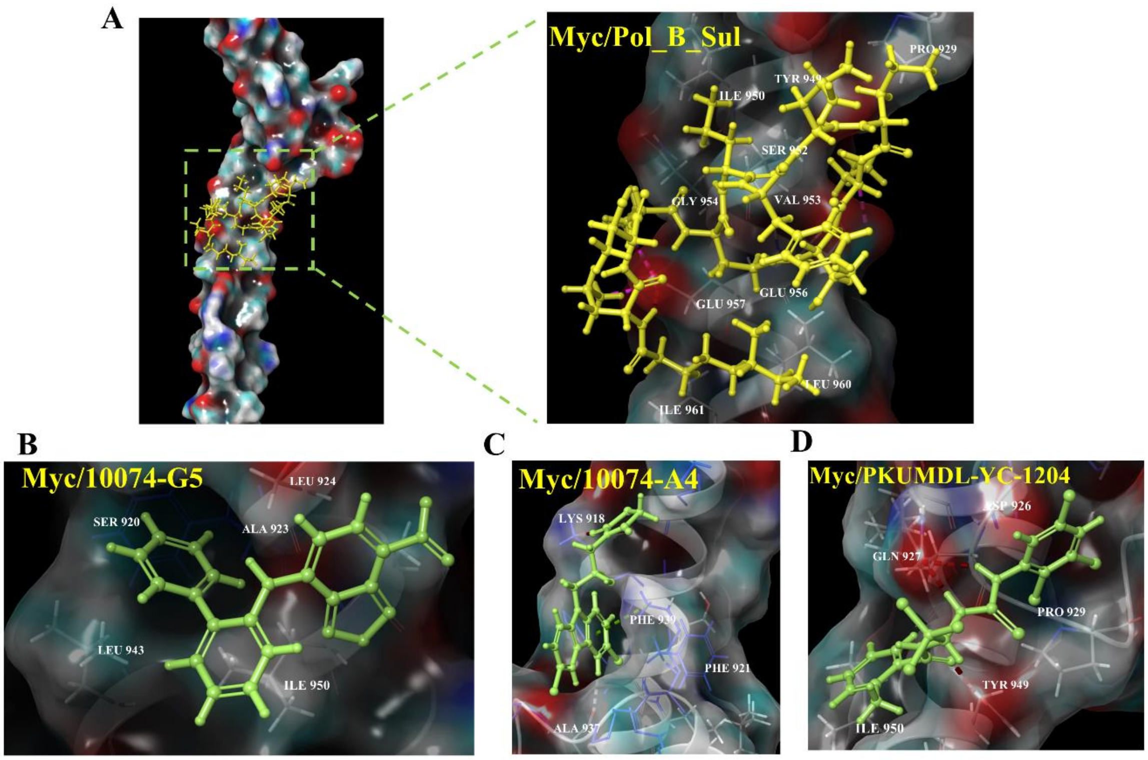

Figure S2. High-throughput virtual screening aided in the discovery of hit bioactive compounds against oncogenic Myc. (A) The potential binding poses and respective ligand molecular interactions of second hit molecule, Pol_B_Sul, and reported Myc inhibitors, 10074-G5 (B) 10074-A4 (C) and PKUMDL-YC-1204 (D) with the identified Site-3 residues of Myc.

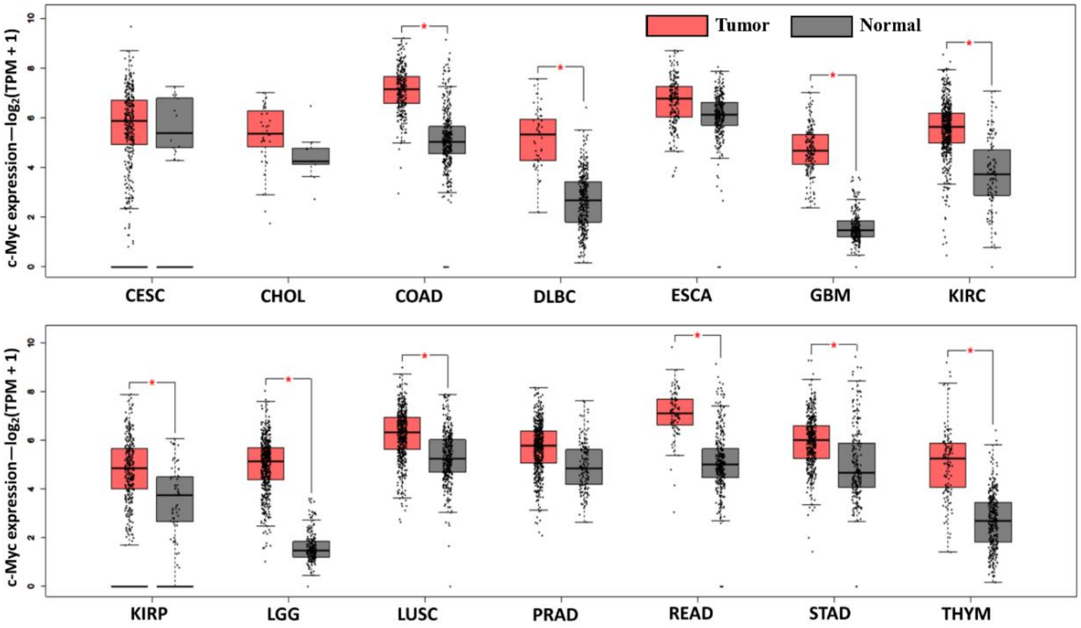

Figure S3. Gene Expression Profiling Interactive Analysis (GEPIA) was performed to validate Myc mRNA expression in various cancer samples vs. normal samples. Data represented as mean ± SD (*$p < 0.05$). (Abbreviation: CECS-Cervical squamous cell carcinoma and endocervical adenocarcinoma, CHOL-Cholangiocarcinoma, COAD-Colon adenocarcinoma, DLBC-Lymphoid Neoplasm Diffuse Large B-cell Lymphoma, ESCA- Esophageal carcinoma, GBM- Glioblastoma multiforme, KIRC- Kidney renal clear cell carcinoma, KIRP- Kidney renal papillary cell carcinoma, LGG- Brain Lower Grade Glioma, LUSC- Lung squamous cell carcinoma, PRAD- Prostate adenocarcinoma, READ- Rectum adenocarcinoma, STAD- Stomach adenocarcinoma, and THYM-Thymoma)

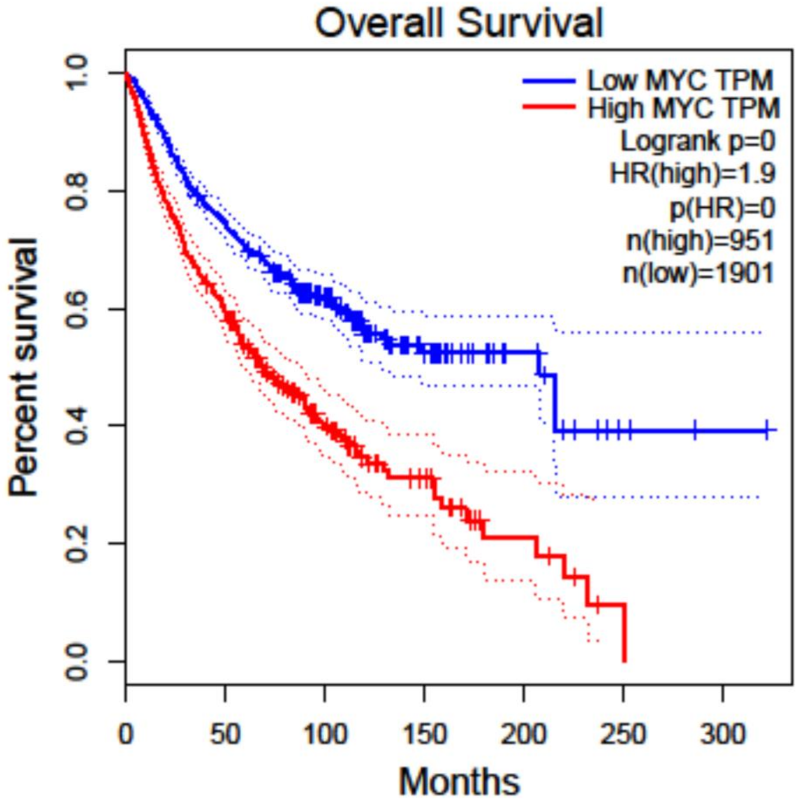

Figure S4. Mantel-Cox survival curve represents high Myc expression with reduced overall survival in different cancer types compared with low Myc expression.

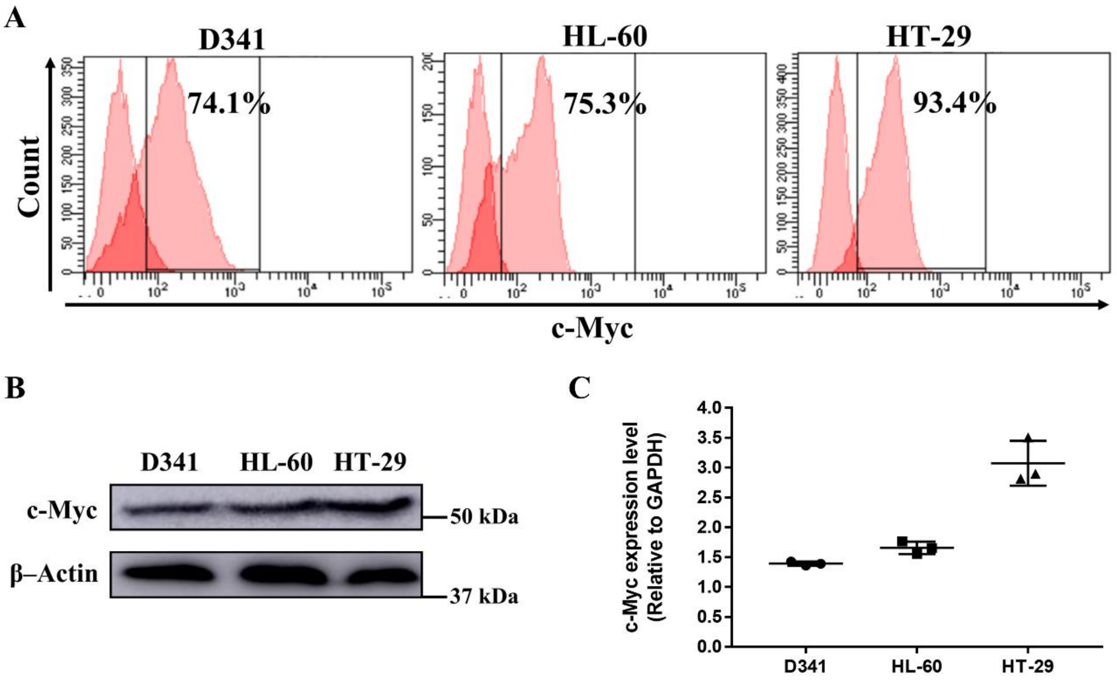

Figure S5. Endogenous expression of Myc in three cell lines viz. D341, HL-60, and HT-29 were characterized by (A) flow cytometry, (B) western blot, and (C) real-time quantitative PCR.

A

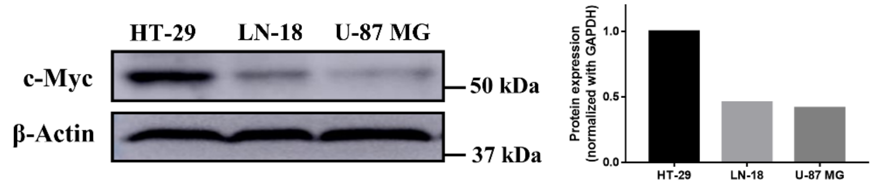

B

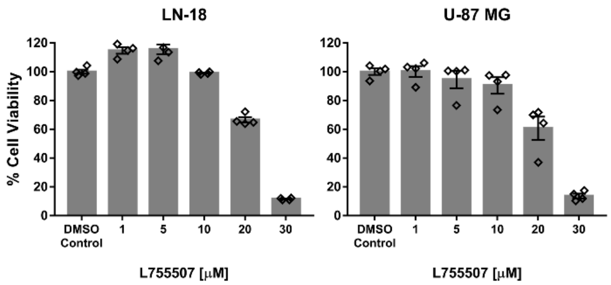

C

IC_{50}	L755507
**LN-18**	22.15 ±2.48μM
**U-87 MG**	21.48 ±1.72μM

Figure S6. Cytotoxicity result of L755507 on low-Myc expressing cell lines. (A) Endogenous expression of Myc on two glioma cell lines, i.e., LN-18 and U-87 MG was probed with western blot and compared with HT-29. (B) Anticancer profile of L755507 on LN-18 and U-87 MG cells after 48 hrs of treatment. The bar represents mean ± SEM of four independent experiments. (C) The obtained IC50 values of L755507 against the two low-Myc expressing cell lines. Values represent mean ± SEM of four independent experiments.

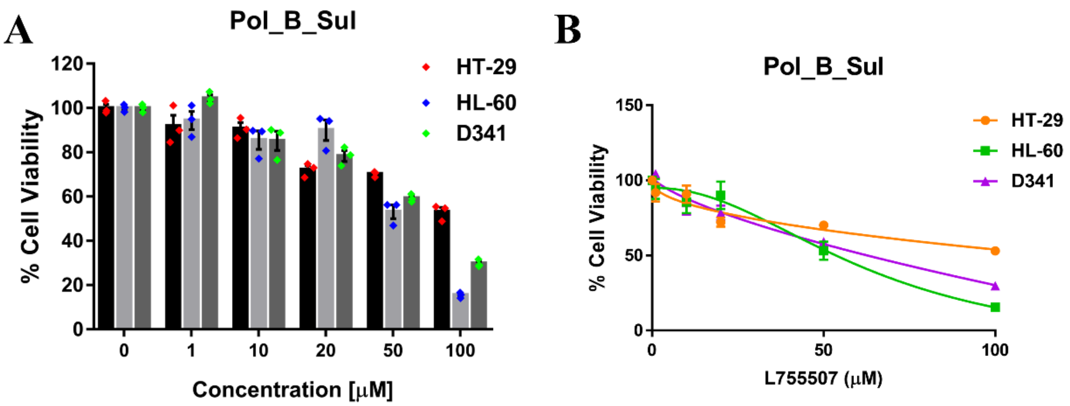

C

IC_{50}	HT-29	HL-60	D341
**Pol_B_Sul**	136.20 ±0.56µM	53.74 ±0.95µM	63.11 ±1.03µM

Figure S7. Cytotoxicity result of the Pol_B_Sul on three Myc expressing cell lines. (A) Anticancer profile of Pol_B_Sul on three cell lines. The bar represents mean ± SEM of three independent experiments. (B) The dose-response curve used to generate the IC_{50} value of Pol_B_Sul on three studied cell lines. Data points represent mean ± SEM of three independent experiments. (C) The obtained IC50 values of Pol_B_Sul. Values represent mean ± SEM of three independent experiments.

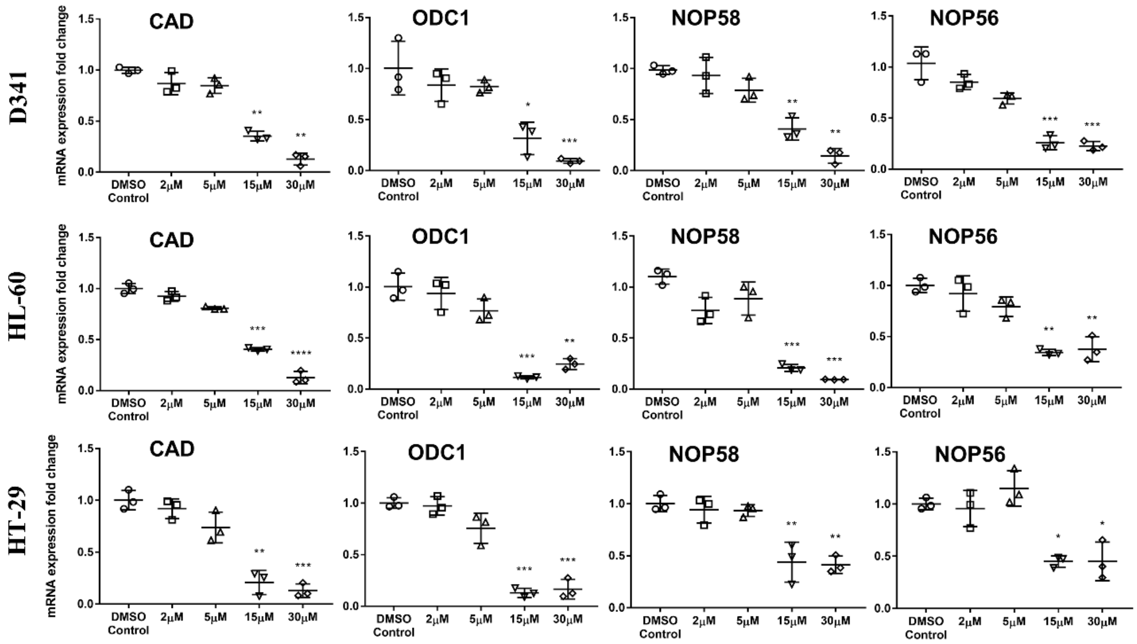

Figure S8. L755507 treatment resulted in the inhibition of Myc transcriptional activity, which is evident from the decreased mRNA level of Myc target genes (CAD, ODC1, NOP58, and NOP56). All three cell lines were treated with the indicated concentration of L755507 for 36 hrs and subjected to qPCR. Plots represent mean ± SD of three experimental replicates (p-value vs DMSO control. *p<0.05, **p<0.01, ***p<0.001, ****p<0.0001).

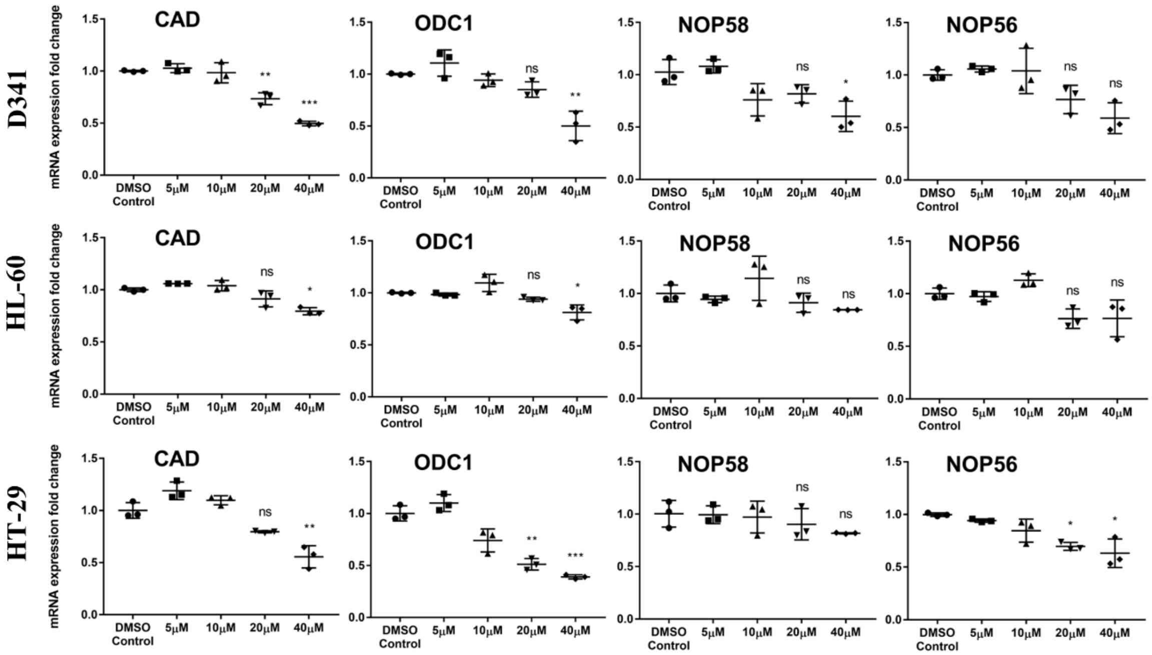

Figure S9. mRNA expression level of Myc target gene (CAD, ODC1, NOP58, and NOP56) after treatment with various concentration of 10074-G5. All three cell lines were treated with the indicated concentration of 10074-G5 for 36 hrs and subjected to qPCR. Plots represent mean ± SD of three experimental replicates (p-value vs DMSO control. ns = non-significant, *p<0.05, **p<0.01, ***p<0.001).

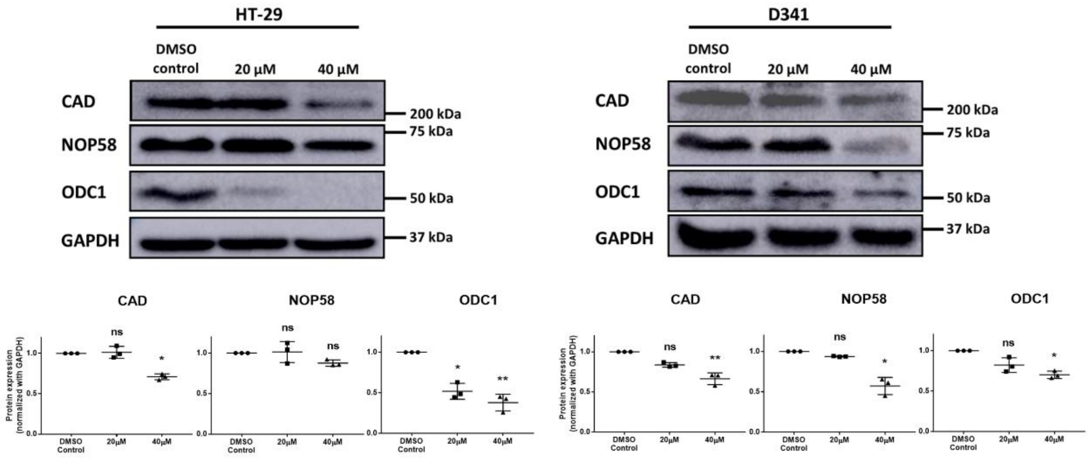

Figure S10. Change in the protein level of c-Myc target genes (CAD, NOP58, and OD1) after treatment with 10074-G5. Plots represent mean ± SD of three independent experiments (p-value vs DMSO control. ns = non-significant *p<0.05, **p<0.01).

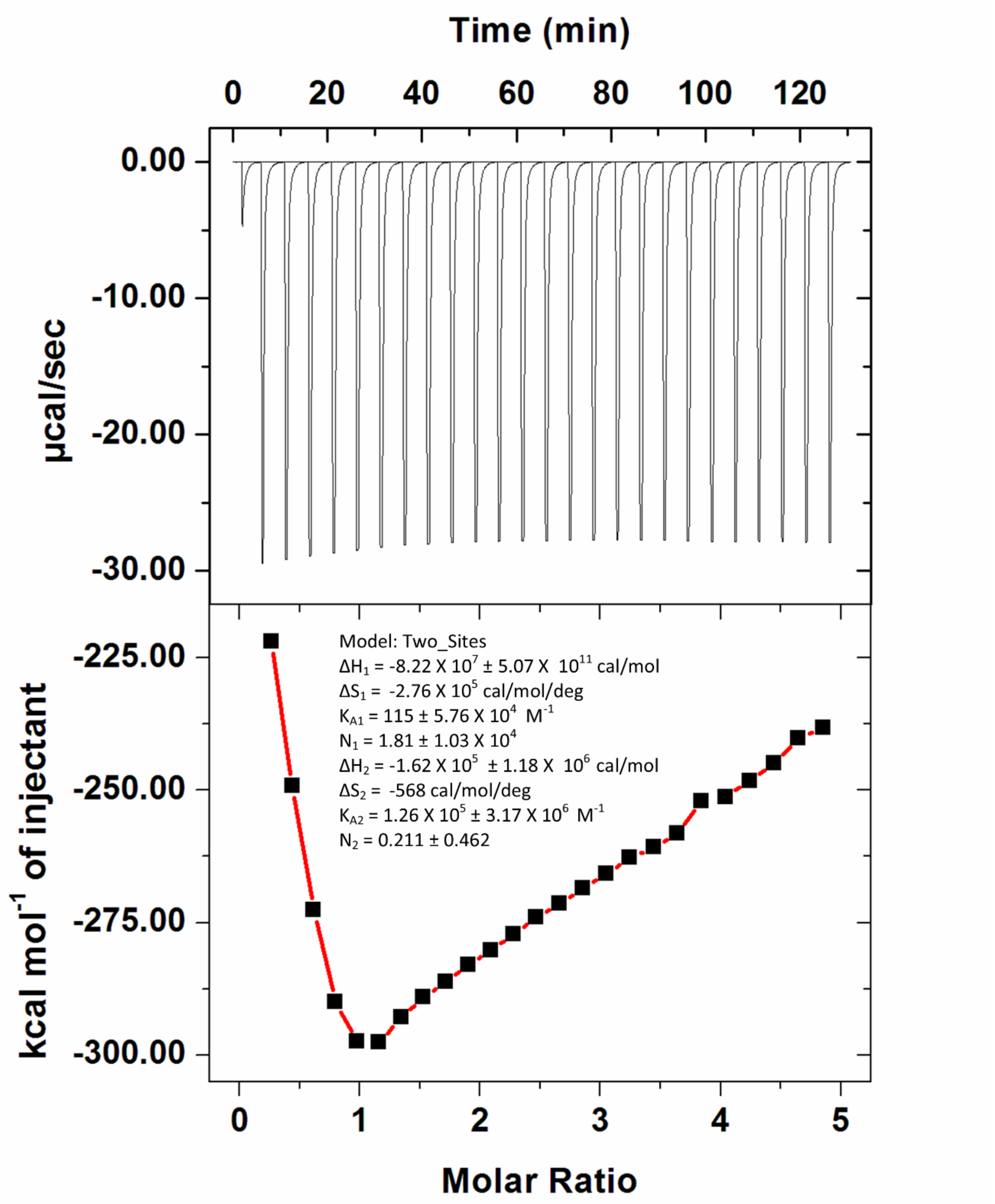

Figure S11. Isothermal titration calorimetry isotherm for the interaction of L755507 with Myc peptide. The upper panel relates to calorimetric response as successive injections of L755507 into sample cell containing Myc peptide. The bottom panel corresponds to integrated heats of interactions as a function of [L755507-Myc] molar ratio.

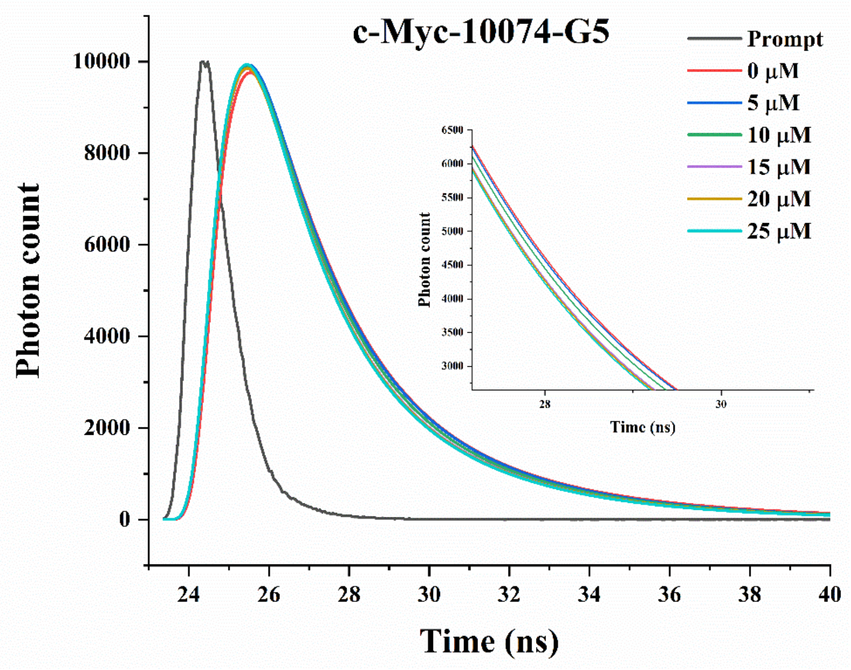

Figure S12. The graph represents the fluorescence lifetime decay measurement of Myc with different concentrations of the ligand 10074-G5.

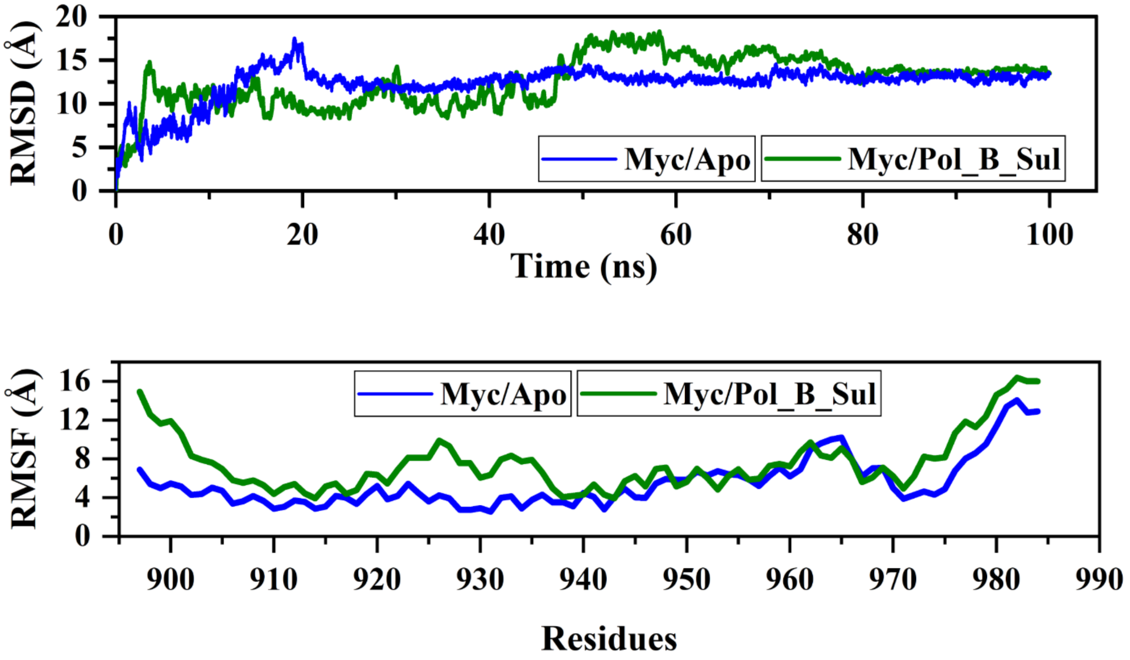

Figure S13. Molecular dynamics simulations analysis of Myc/Pol_B_Sul complex for 100 ns. Root Mean Square Deviation (RMSD) of the C-a atoms of unbound Myc (blue) and Myc bound to Pol_B_Sul (olive) and Root Mean Square Fluctuation (RMSF) in C-a atoms of unbound Myc (blue) and Myc bound to Pol_B_Sul (olive).

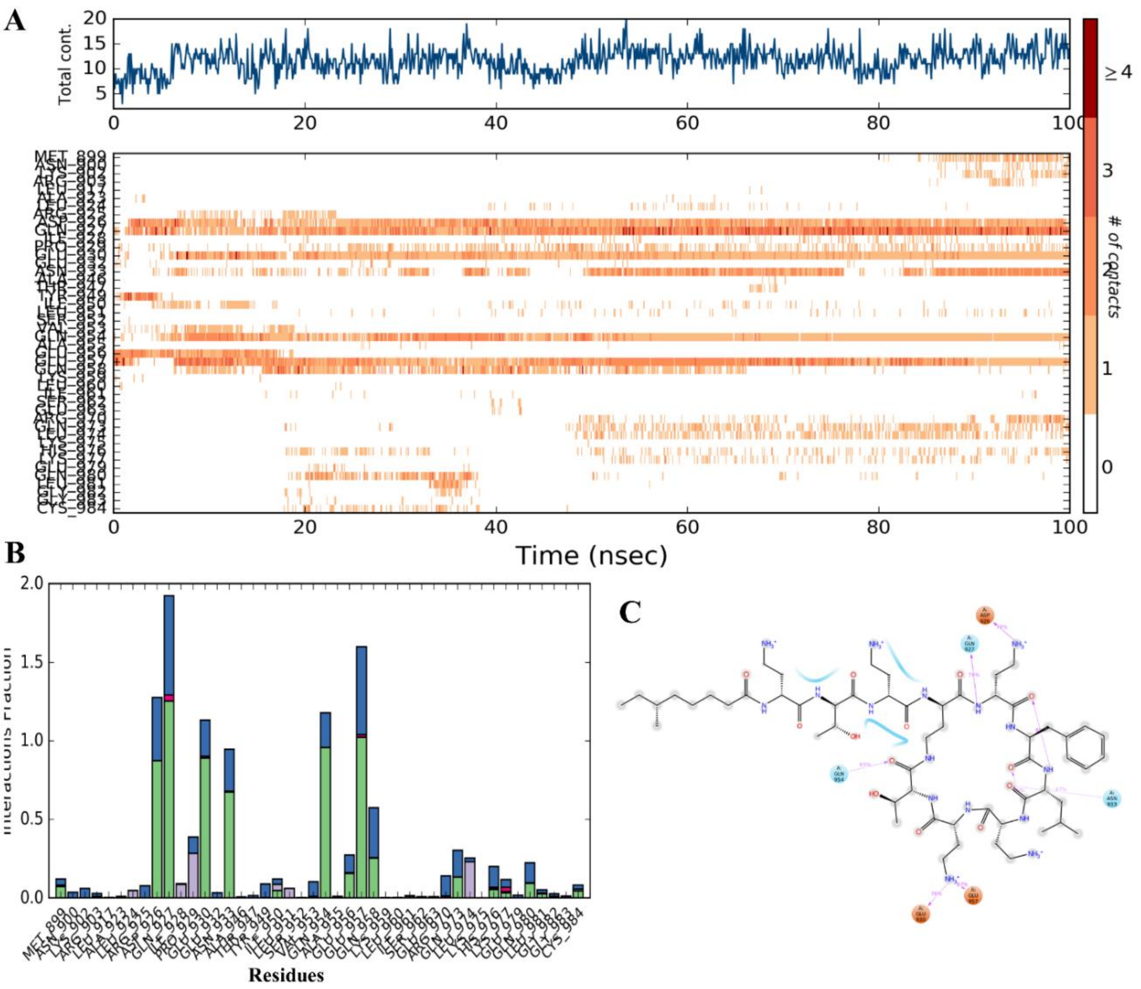

Figure S14. (A) Timeline depiction of the total number of specific contacts the Myc made with Pol_B_Sul throughout the simulation period of 100ns. (B) Histogram representation of various interactions formed by residues of Myc with Pol_B_Sul (values more than 1 show multiple interactions) during a course of computational simulations. The color in the bars, i.e., green, purple, red, and blue, represents hydrogen bonds, hydrophobic, ionic, and water bridge interactions. (C) Interaction occupancy of Site-3 residues with Pol_B_Sul throughout the simulation period.

Table S1. Complete detail of antibodies used in different experiments in this study.

**Name**	**Supplier**	**Catalog Number**	**Application**
Anti-Myc-FITC	Miltenyi Biotech	130-116-653	Flow cytometry
Mouse-IgG1-FITC (Isotype)	Miltenyi Biotech	130-113-761	Flow cytometry
CAD Polyclonal Antibody	Thermo fischer scientific	PA5-71471	Western blot
ODC1 Monoclonal Antibody	Thermo fischer scientific	MA5-25138	Western blot
NOP58 Polyclonal Antibody	Thermo fischer scientific	PA5-53811	Western blot
MAX Polyclonal Antibody	Thermo fischer scientific	PA5-80800	Western blot
Myc Monoclonal Antibody	Thermo fischer scientific	13-2500	Western blot/Co-immunoprecipitation
Anti-beta-Actin Monoclonal Antibody	Sigma-Aldrich	MABT825	Western blot
GAPDH Monoclonal Antibody	Thermo fischer scientific	AM4300	Western blot
Goat anti-Rabbit IgG (H+L) Secondary antibody	Thermo fischer scientific	65-6120	Western blot
Goat anti-Mouse IgG (H+L) Secondary antibody	Thermo fischer scientific	62-6520	Western blot

Table S2. List of Real-time quantitative PCR primers used in the study.

	Gene	Forward Primer 5'-3'	Reverse Primer 5'-3'
1	Actβ	GAGCACAGAGCCTCGCCTTT	ACATGCCGGAGCCGTTGTC
2	GAPDH	TGCACCACCAACTGCTTAGC	GGCATGGACTGTGGTCATGAG
3	Myc	AATGAAAAGGCCCCCAAGGTAGTTATCC	GTCGTTTCCGCAACAAGTCCTCTTC
4	CAD	TAGTCCTTGGCTCTGGCGTCTA	TAGTCGGTGCTGACTGTCTCTG
5	ODC1	CCAAAGCAGTCTGTCGTCTCAG	CAGAGATTGCCTGCACGAAGGT
6	NOP56	GGCTAAGGCTATTCTGGATGCC	TGTGTAGGCTCTGGCGGTATTC
7	NOP58	TGGTATGGCTGGCATTTCCCTG	GCAGCAACTCAGAAAGCTTGGC

Table S3. Predicted shallow active sites on the apo structure of Myc from the Myc-MAX complex generated by SiteMap.

**Sites**	**Site Score**	**Draggability score**	**Solvent Exposure**	**Hydrophobicity**	**Hydrophilicity**	**Residues**
Site 1	0.857	0.963	0.783	0.055	0.572	911,914,915,917,918, 921,922,925,935,936, 937,938,939,942
Site 3	0.744	0.861	0.89	0.58	0.14	913,917,920,921,924, 927,928,943,946,947, 949,950,953
Site 2	0.695	0.779	0.855	0.071	0.435	925,926,929,931,932, 933,934,935
Site 4	0.677	0.776	0.903	0.343	0.2	960,961,963,964,967, 968,970,971,974,975
Site 5	0.631	0.712	0.889	0.143	0.31	963,966,967,969,970, 973,974,976,977,980

Table S4. Docking score and binding energy of hit bioactive compounds and the reported Myc inhibitors bind to the identified site on Myc.

**Compound**	**Docking score (kcal/mol)**	**MMGBSA ΔG Bind (kcal/mol)**	**2D Structure**
L755507	-7.151	-54.925	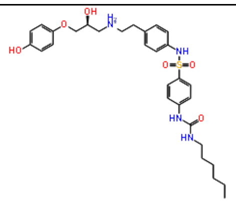
Salvianolic acid B	-12.886	-50.504	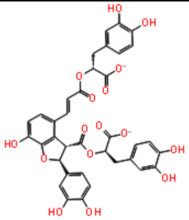
Sennoside A	-7.993	-50.108	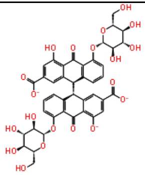
Paromomycin Sulfate	-7.152	-42.81	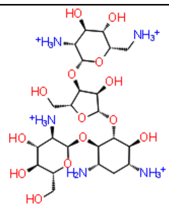
Heparin sodium	-8.956	-42.277	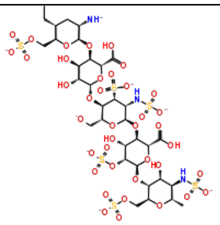
Pemetrexed	-7.648	-39.066	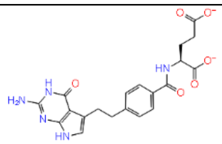
Polymyxin B sulphate	-9.584	-38.927	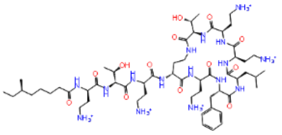

Rosmarinic acid	-7.772	-37.905	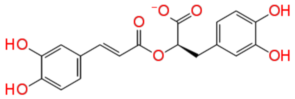
Acarbose	-7.426	-37.342	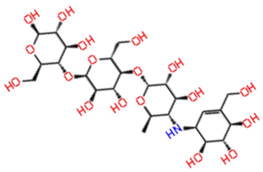
Cefixime	-7.028	-37.167	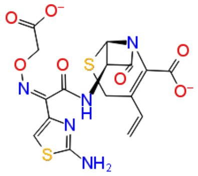
Neomycin sulfate	-7.002	-36.528	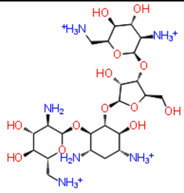
Leucovorin Calcium	-7.76	-36.409	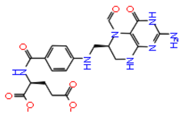
Omniscan (gadodiamide)	-7.373	-36.315	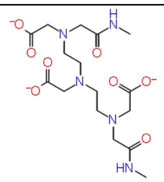
Folic Acid	-7.508	-36.116	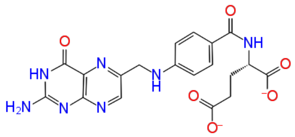
Raltitrexed	-8.86	-35.47	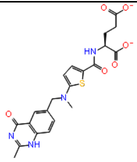

**PKUMDL-YC-1204***	**-2.651**	**-37.294**	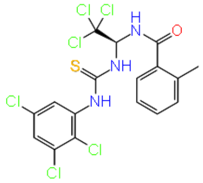
**10074-G5***	**-1.29**	**-36.902**	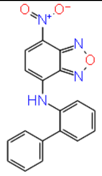
**10074-A4***	**-2.155**	**-21.515**	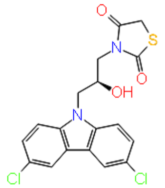

***Reported Myc/MAX inhibitors from the literature.**

Table S5: List of sub-structures from PubChem database of L755507 with their docking score and binding energy scores against identified Site-3 of Myc.

**PubChem Compound ID**	**2D structure**	**IUPAC Name**	**Docking score (kcal/mol)**	**MMGBSA ΔG Bind (kcal/mol)**
L755507	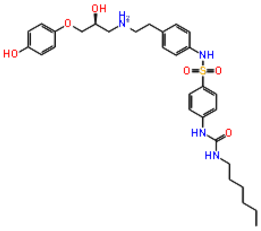	1-hexyl-3-[4-[[4-[2-[[(2S)-2-hydroxy-3-(4-hydroxyphenoxy)propyl]amino]ethyl]phenyl]sulfamoyl]phenyl]urea	-7.151	-54.925
44268498	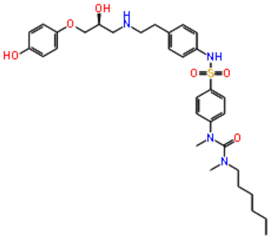	1-hexyl-3-[4-[[4-[2-[[(2S)-2-hydroxy-3-(4-hydroxyphenoxy)propyl]amino]ethyl]phenyl]sulfamoyl]phenyl]-1,3-dimethylurea	-6.583	-61.607
44268569	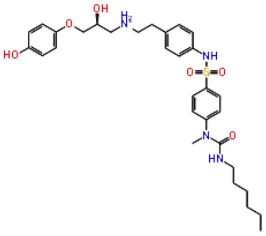	3-hexyl-1-[4-[[4-[2-[[(2S)-2-hydroxy-3-(4-hydroxyphenoxy)propyl]amino]ethyl]phenyl]sulfamoyl]phenyl]-1-methylurea	-6.374	-37.949

18683394	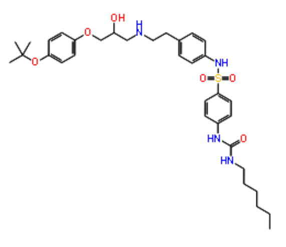	1-hexyl-3-[4-[[4-[2-[[2-hydroxy-3-[4-[(2-methylpropan-2-yl)oxy]phenoxy]propyl]amino]ethyl]phenyl]sulfamoyl]phenyl]urea	-6.248	-48.003
46882763	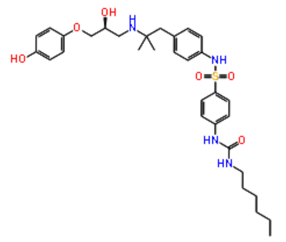	1-hexyl-3-[4-[[4-[2-[[(2S)-2-hydroxy-3-(4-hydroxyphenoxy)propyl]amino]-2-methylpropyl]phenyl]sulfamoyl]phenyl]urea	-6.066	-37.189
44268571	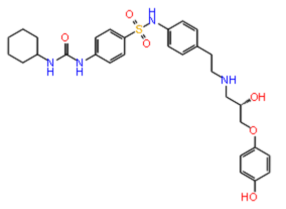	1-cyclohexyl-3-[4-[[4-[2-[[(2S)-2-hydroxy-3-(4-hydroxyphenoxy)propyl]amino]ethyl]phenyl]sulfamoyl]phenyl]urea	-5.661	-43.631
59879613	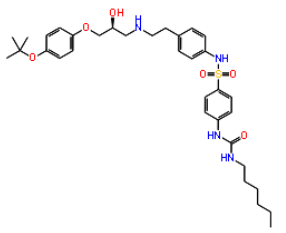	1-hexyl-3-[4-[[4-[2-[[(2S)-2-hydroxy-3-[4-[(2-methylpropan-2-yl)oxy]phenoxy]propyl]amino]ethyl]phenyl]sulfamoyl]phenyl]urea	-4.989	-40.572
44268495	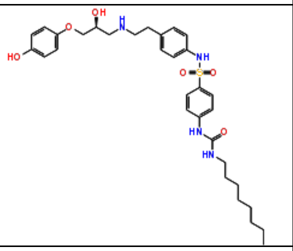	1-[4-[[4-[2-[[(2S)-2-hydroxy-3-(4-hydroxyphenoxy)propyl]amino]ethyl]phenyl]sulfamoyl]phenyl]-3-octylurea	-4.974	-59.717
10122287	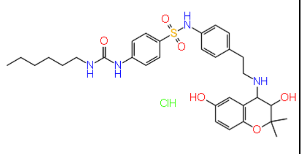	1-[4-[[4-[2-[(3,6-dihydroxy-2,2-dimethyl-3,4-dihydrochromen-4-yl)amino]ethyl]phenyl]sulfamoyl]phenyl]-3-hexylurea;hydrochloride	-3.061	-53.202

10122288	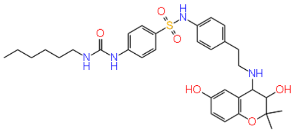	1-[4-[[4-[2-[(3,6-dihydroxy-2,2-dimethyl-3,4-dihydrochromen-4-yl)amino]ethyl]phenyl]sulfamoyl]phenyl]-3-hexylurea	-3.061	-53.205

Table S6: Prediction of pharmacokinetic properties of L755507 using QikProp and OSIRIS DataWarrior programs. a: Estimated number of H-bond donors; b: Estimated number of H-bond acceptors; c: Predicted octanol/water partition coefficient; d: Predicted aqueous solubility; e: Conformation-independent predicted aqueous solubility.

**QikProp v5.2**							
**Molecule**	**Mol. Wt (130.0 - 725.0)**	**H-donors[a] (0.0 - 6.0)**	**H-acceptors[b] (2.0 - 20.0)**	**LogP[c] (-2.0 - 6.5)**	**LogS[d] (-6.5 – 0.5)**	**CIQPlogS[e] (-6.5 – 0.5)**	
L755507	584.73	6	11.2	3.232	-6.303	-5.6	
**OSIRIS DataWarrior**							
**Molecule**	**Mol. Wt**	**H-donors[a]**	**H-acceptors[b]**	**LogP[c]**	**LogS[d]**	**Mutagenic**	**Tumorigenic**
L755507	585.74	6	10	2.689	-5.663	None	None

Movie S1 (separate file). 100ns simulation trajectory of Myc/L755507 complex showed a stable binding of compound (Red) with Myc (Blue).

References

1. Yin, X., Giap, C., Lazo, J. S., and Prochownik, E. V. (2003) Low molecular weight inhibitors of Myc-Max interaction and function. *Oncogene*. **22**, 6151–6159
2. Yu, C., Niu, X., Jin, F., Liu, Z., Jin, C., and Lai, L. (2016) Structure-based Inhibitor Design for the Intrinsically Disordered Protein c-Myc. *Sci. Rep.* **6**, 1–11
